# Identifying a biological signature of prenatal maternal stress

**DOI:** 10.1172/jci.insight.143007

**Published:** 2021-01-25

**Authors:** James M. Keane, Ali S. Khashan, Fergus P. McCarthy, Louise C. Kenny, James M. Collins, Sarah O’Donovan, Jillian Brown, John F. Cryan, Timothy G. Dinan, Gerard Clarke, Siobhain M. O’Mahony

**Affiliations:** 1 APC Microbiome Ireland and; 2 School of Public Health, University College Cork, Ireland.; 3 The Irish Centre for Maternal and Child Health Research (INFANT), Cork University Maternity Hospital, Cork, Ireland.; 4 Department of Obstetrics and Gynaecology, University College Cork, Cork, Ireland.; 5 Department of Women’s and Children’s Health, Institute of Translational Medicine, University of Liverpool, United Kingdom.; 6 Department of Anatomy and Neuroscience and; 7 Department of Psychiatry and Neurobehavioral Science, University College Cork, Cork, Ireland.

**Keywords:** Inflammation, Neuroscience, Obstetrics/gynecology

## Abstract

Psychological stress affects maternal gastrointestinal (GI) permeability, leading to low-grade inflammation, which can negatively affect fetal development. We investigated a panel of circulating markers as a biological signature of this stress exposure in pregnant women with and without the stress-related GI disorder irritable bowel syndrome (IBS). Markers of GI permeability and inflammation were measured in plasma from healthy and IBS cohorts of women at 15 and 20 weeks’ gestation. Biomarkers were evaluated with respect to their degree of association to levels of stress, anxiety, and depression as indicated by responses from the Perceived Stress Scale, State-Trait Anxiety Inventory, and Edinburgh Postnatal Depression Scale. High levels of stress were associated with elevations of soluble CD14, lipopolysaccharide binding protein (LBP), and tumor necrosis factor–α, while anxiety was associated with elevated concentrations of C-reactive protein (CRP) in otherwise healthy pregnancies. Prenatal depression was associated with higher levels of soluble CD14, LBP, and CRP in the healthy cohort. High levels of prenatal anxiety and depression were also associated with lower concentrations of tryptophan and kynurenine, respectively, in the IBS cohort. These markers may represent a core maternal biological signature of active prenatal stress, which can be used to inform intervention strategies via stress reduction techniques or other lifestyle approaches. Such interventions may need to be tailored to reflect underlying GI conditions, such as IBS.

## Graphical abstract

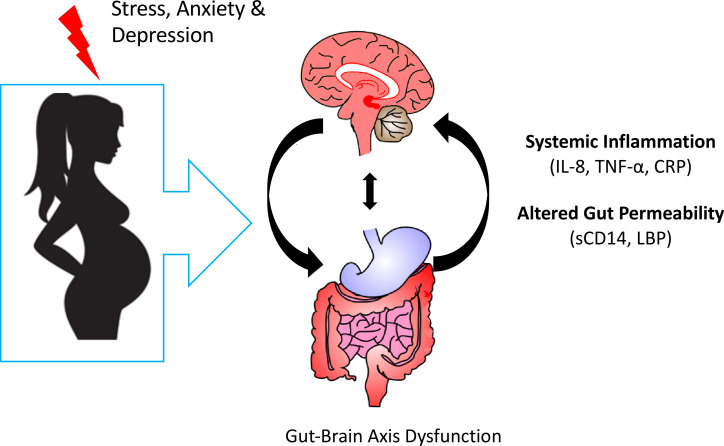

## Introduction

Maternal prenatal stress is associated with several unfavorable pregnancy outcomes, including preterm birth (1) and low infant birth weight (2), while anxiety and depression early in pregnancy are considered risk factors for preeclampsia, a potentially fatal complication of pregnancy (3). Prenatal stress is also associated with altered programming of the developing fetal brain and an increased likelihood of behavioral problems manifesting during childhood and diagnosis of psychopathology in later life (4). Preclinical studies have demonstrated that induction of prenatal stress impedes optimum cognitive (5), behavioral (6), and psychosocial (7) development in the offspring. These findings are supported by prospective studies in humans, with neurodevelopmental ramifications encompassing temperamental and behavioral difficulties (8), cognitive impairments (9), and a hyperactive stress response along the hypothalamic-pituitary-adrenal axis (10).

Delineation of the molecular pathways that link prenatal stress to neurodevelopment is a critical first step in identifying biological risk factors associated with adverse outcomes. Conventional pathways of translocation of maternal glucocorticoids (11) and proinflammatory cytokines (12) across the placenta and their direct action on the developing fetal brain have been the focus of many mechanistic studies in this area. It is becoming increasingly apparent, however, that alternative indirect routes may be more relevant to eliciting the downstream effects of prenatal stress, via neuroimmune interactions along the microbiota-gut-brain axis (13).

A loss of gastrointestinal (GI) barrier integrity allows bacterial components, such as lipopolysaccharides (LPS) contained in the cell walls of gram-negative bacteria, to migrate into the general circulation and contribute to a systemic proinflammatory state (14). In confirmation of this, functional and/or compositional alterations in the gut microbiota modify intestinal barrier permeability (15). Concomitantly, acute psychological stress has been shown to increase permeability of the small intestine (16), whereas disruption of the intestinal mucosal barrier has been implicated in the inflammatory pathophysiology of depression (17). The state of chronic, low-grade inflammation that results from increased gut permeability is also known to negatively affect neurodevelopment (18).

A mechanistic pathway implicated in this negative impact is the alteration in tryptophan availability due to increased degradation of this essential amino acid along the kynurenine pathway (19, 20). Altered gut microbiota profile, elevated gut permeability, low-grade inflammation, and tryptophan degradation along the kynurenine pathway are all traits characteristic of irritable bowel syndrome (IBS), a highly prevalent functional disorder of the GI tract (21–24). Notably, stress is implicated in the pathogenesis of IBS, with its trademark symptoms, including intestinal sensitivity, motility, and permeability as well as mucosal immune activation, each shown to be exacerbated in the presence of psychological stresses (25). It is also well established that rates of psychiatric disorders, particularly anxiety and depression, are elevated among patients with IBS (26, 27).

Here we investigate whether GI permeability and systemic inflammation are elevated in mothers who experience higher levels of stress, anxiety, and depression during pregnancy and whether this stress signature is potentially augmented in women with IBS. We investigated circulating markers of these pathophysiological functions in the first and second trimester of pregnancy to identify associations that may serve as biological indicators of active prenatal maternal stress in a healthy cohort and in women with IBS. We anticipate that an assemblage of molecular indicators related to these processes, whose augmentation point to adverse pregnancy and neurodevelopmental outcomes, will aid in screening for at-risk pregnant women and informing appropriate intervention strategies aimed at counteracting the effects of prenatal maternal stress via either stress reduction techniques or other lifestyle approaches.

## Results

### Healthy cohort associations

#### Associations between maternal perceived stress and biomarker levels in the healthy cohort.

**Figure 1:**
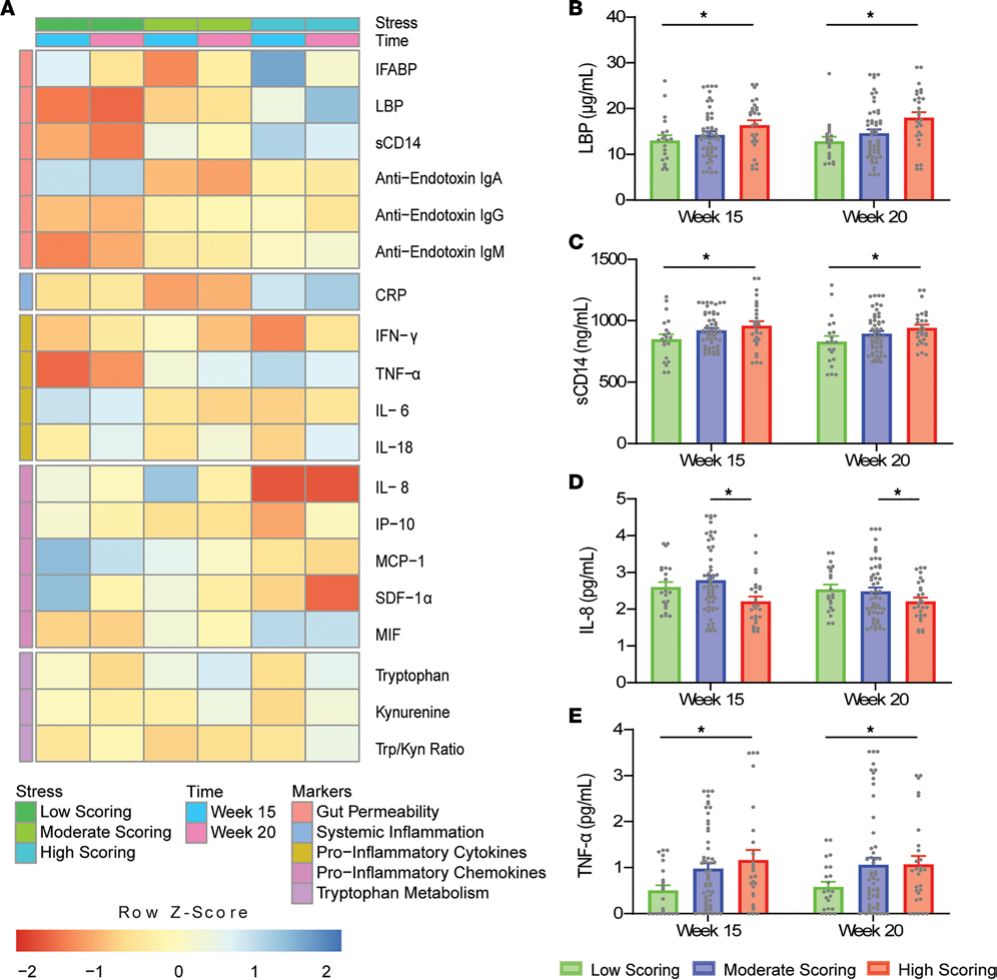
Differences in PSS-scoring groups across gestational time points in the healthy cohort (*N* = 22/54/28 from low/moderate/high). (**A**) Heatmap of normalized biomarker means across scoring groups and time points. Individual boxes are represented on a scale from red to blue. Darker red boxes represent markers whose concentrations were found to be at least 1 standard deviation lower than the mean concentration across stress group and time point. Darker blue boxes represent markers whose concentrations were found to be at least 1 standard deviation higher than the mean concentration across stress group and time point. Lower *z* scores are evident for lipopolysaccharide binding protein (LBP), soluble CD14 (sCD14), and TNF-α in the low-scoring group at 15 and 20 weeks; and for IL-8 in the high-scoring group at 15 and 20 weeks. Significant differences between PSS-scoring groups are illustrated for (**B**) LBP, (**C**) sCD14, (**D**) IL-8, and (**E**) TNF-α (2-way ANOVA with Tukey’s honestly significant differences test, HSD). *P < 0.05.

Significant differences in Perceived Stress Scale (PSS) scoring groups across gestational time points are outlined in Figure 1. Soluble CD14 (sCD14), lipopolysaccharide binding protein (LBP), and TNF-α levels were found to significantly increase in the high-scoring group (110.96 ± 43.5 ng/mL, *P* = 0.033; 4.27 ± 1.57 μg/mL, *P* = 0.021; 0.62 ± 0.26 pg/mL, *P* = 0.049; respectively) compared with the low-scoring group. Conversely, IL-8 concentrations were observed to be significantly lower in the high-scoring group (–0.45 ± 0.16 pg/mL, *P* = 0.018) compared with moderate scorers. Significant differences in biomarker levels between scoring groups were not complemented by the presence of linear associations between circulating biomarkers’ concentrations and PSS scores in healthy participants, as outlined in Supplemental Table 4; supplemental material available online with this article; https://doi.org/10.1172/jci.insight.143007DS1

#### Associations between maternal anxiety and biomarker levels in the healthy cohort.

**Figure 2:**
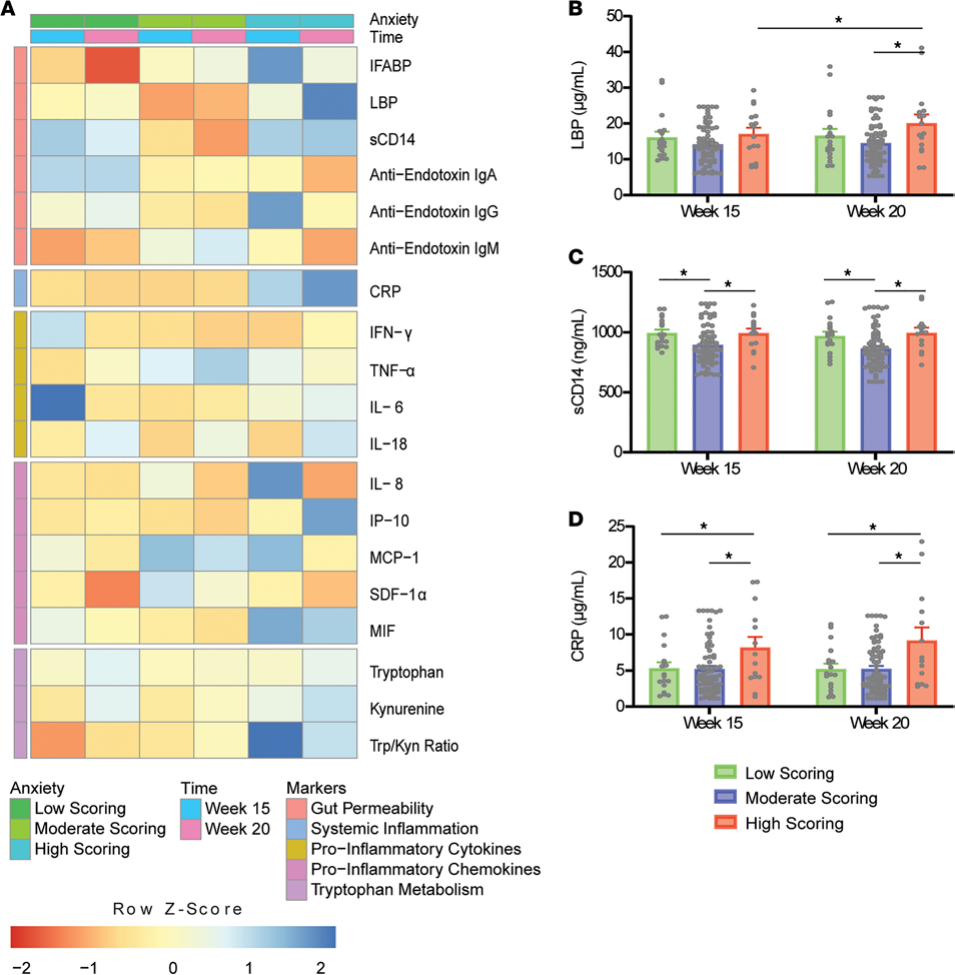
Differences in STAI-scoring groups across gestational time points in the healthy cohort (*N* = 18/70/16 from low/moderate/high). (**A**) Heatmap of normalized biomarker means across scoring groups and time points. Individual boxes are represented on a scale from red to blue. Darker red boxes represent markers whose concentrations were found to be greater than 1 standard deviation lower than the mean concentration across stress group and time point. Darker blue boxes represent markers whose concentrations were found to be greater than 1 standard deviation higher than the mean concentration across stress group and time point. Lower *z* scores are evident for intestinal fatty acid binding protein (IFABP) in the low-scoring group at 20 weeks. Higher *z* scores are evident for IL-6 in the low-scoring group at 15 weeks; for IFABP, anti-endotoxin IgG, IL-8, and tryptophan/kynurenine ratio (Trp/Kyn ratio) in the high-scoring group at 15 weeks; and for LBP, C-reactive protein (CRP), and IFN-γ–induced protein 10 (IP-10) in the high-scoring group at 20 weeks. Significant differences between STAI-scoring groups are illustrated for (**B**) LBP, (**C**) sCD14, and (**D**) CRP (2-way ANOVA with Tukey’s HSD). *P < 0.05. MCP-1, monocyte chemoattractant protein-1; MIF, macrophage migration inhibitory factor; SDF-1α, stromal cell-derived factor 1α.

Significant differences in State-Trait Anxiety Inventory (STAI) scoring groups across gestational time points are outlined in Figure 2. sCD14 levels were found to significantly increase in the high- (113.02 ± 42.46 ng/mL, *P* = 0.025) and low-scoring groups (100.72 ± 38.36 ng/mL, *P* = 0.027) compared with moderate scorers. LBP levels were also observed to increase with high (5.56 ± 1.95 μg/mL, *P* = 0.015) compared with moderate scores for measurements taken at 20 weeks’ gestation, with concentrations significantly increasing from 15 weeks’ gestation (3.01 ± 1.14 μg/mL, *P* = 0.019) for high-scoring participants only. Meanwhile, CRP levels were found to be significantly higher in the high-scoring group compared with moderate- (3.43 ± 1.09 μg/mL, *P* = 0.006) and low-scoring groups (3.39 ± 1.33 μg/mL, *P* = 0.032). Significant differences in LBP and CRP levels between scoring groups were complemented by the presence of positive linear associations between their circulating concentrations and STAI scores in both simple and adjusted models, as outlined in Supplemental Table 5. Additional positive associations with STAI scores are noted for simple and adjusted models of the Trp/Kyn ratio at 15 weeks’ gestation, as well as for IL-6 and covariate-adjusted IP-10 and Trp/Kyn ratio at 20 weeks’ gestation.

#### Associations between maternal depression and biomarker levels in the healthy cohort.

**Figure 3:**
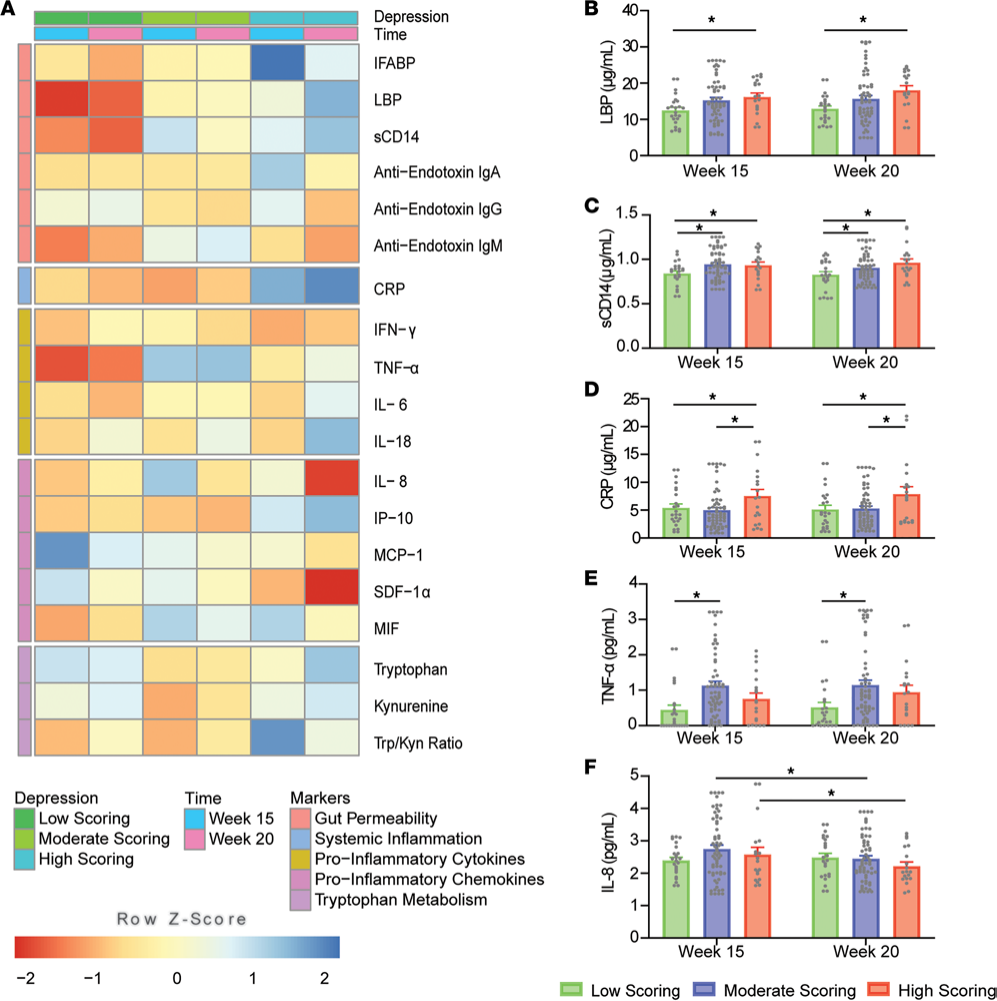
Differences in EPDS scoring groups across gestational time points in the healthy cohort (*N* = 24/61/19 from low/moderate/high). (**A**) Heatmap of normalized biomarker means across scoring groups and time points. Individual boxes are represented on a scale from red to blue. Darker red boxes represent markers whose concentrations were found to be greater than 1 standard deviation lower than the mean concentration across stress group and time point. Darker blue boxes represent markers whose concentrations were found to be greater than 1 standard deviation higher than the mean concentration across stress group and time point. Lower *z* scores are evident for LBP, sCD14, and TNF-α in the low-scoring group at 15 and 20 weeks and for IL-8 and SDF-1α in the high-scoring group at 20 weeks. Higher *z* scores are evident for IFABP and Trp/Kyn ratio in the high-scoring group at 15 weeks and for CRP in the high-scoring group at 15 and 20 weeks. Significant differences between EPDS-scoring groups are illustrated for (**B**) LBP, (**C**) sCD14, (**D**) CRP, (**E**) TNF-α, and (**F**) IL-8 (2-way ANOVA with Tukey’s HSD). *P < 0.05.

Significant differences in Edinburgh Postnatal Depression Scale (EPDS) scoring groups across gestational time points are outlined in Figure 3. sCD14, LBP, and CRP levels were found to significantly increase in the high-scoring group (113.87 ± 47.09 ng/mL, *P* = 0.046; 4.41 ± 1.74 μg/mL, *P* = 0.035; 2.71 ± 1.13 μg/mL, *P* = 0.048; respectively) compared with low scorers, as well as compared with moderate scorers (2.56 ± 0.97 μg/mL, *P* = 0.026) in the case of CRP. Concentrations of sCD14 and TNF-α were also observed to be elevated in the moderate-scoring group (93.02 ± 37.43 ng/mL, *P* = 0.039; 0.66 ± 0.21 pg/mL, *P* = 0.006; respectively) compared with the low-scoring group. Significant differences in LBP levels between scoring groups were in line with the presence of positive linear associations, as outlined in Supplemental Table 6, between its circulating concentrations and EPDS scores observed at 20 weeks’ gestation in both simple and adjusted models. For both moderate- and high-scoring groups, IL-8 levels at 20 weeks’ gestation were noted to be significantly decreased compared with 15 weeks’ gestation (–0.30 ± 0.09 pg/mL, *P* = 0.001; –0.36 ± 0.16 pg/mL, *P* = 0.035; respectively). A negative association was also noted between circulating IL-8 levels and EPDS scores at 20 weeks’ gestation, although this association was no longer significant in the adjusted model.

### IBS cohort associations

#### Associations between maternal perceived stress and biomarker levels in the IBS cohort.

**Figure 4:**
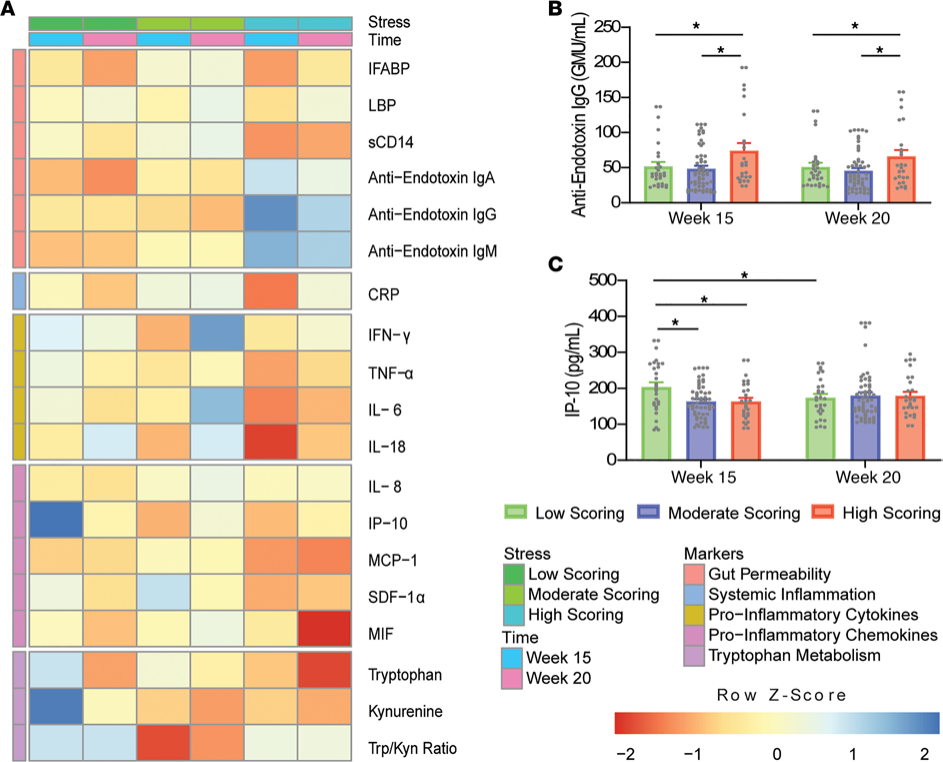
Differences in PSS-scoring groups across gestational time points in the IBS cohort (*N* = 29/55/21 from low/moderate/high). (**A**) Heatmap of normalized biomarker means across scoring groups and time points. Individual boxes are represented on a scale from red to blue. Darker red boxes represent markers whose concentrations were found to be at least 1 standard deviation lower than the mean concentration across stress group and time point. Darker blue boxes represent markers whose concentrations were found to be at least 1 standard deviation higher than the mean concentration across stress group and time point. Higher *z* scores are evident for IP-10 and Kyn in the low-scoring group at 15 weeks and for anti-endotoxin IgG in the high-scoring group at 15 weeks. Significant differences between PSS-scoring groups are illustrated for (**B**) anti-endotoxin IgG and (**C**) IP-10 (2-way ANOVA with Tukey’s HSD). *P < 0.05. GMU, IgG median units.

Significant differences in PSS scoring groups across gestational time points are outlined in Figure 4. Anti-endotoxin IgG levels were found to be significantly higher in the high-scoring group compared with moderate- (30.12 ± 9.15 GMU/mL, *P* = 0.004) and low-scoring groups (25.69 ± 10.22 GMU/mL, *P* = 0.036). This is supported by the presence of a positive association between circulating IgG levels and PSS scores, although this association was no longer significant in the adjusted model. In contrast, IP-10 concentrations were found to be significantly higher in the low-scoring group compared with moderate- (40.10 ± 12.99 pg/mL, *P* = 0.007) and high-scoring groups (39.04 ± 16.23 pg/mL, *P* = 0.047) for measurements taken at 15 weeks’ gestation. However, IP-10 levels at 20 weeks’ gestation were also noted to be significantly decreased compared with 15 weeks’ gestation (–29.56 ± 9.23 pg/mL, *P* = 0.003) in low-scoring participants. No further linear relationships were observed between biomarkers’ concentrations and PSS scores in the IBS cohort.

#### Associations between maternal anxiety and biomarker levels in the IBS cohort.

**Figure 5:**
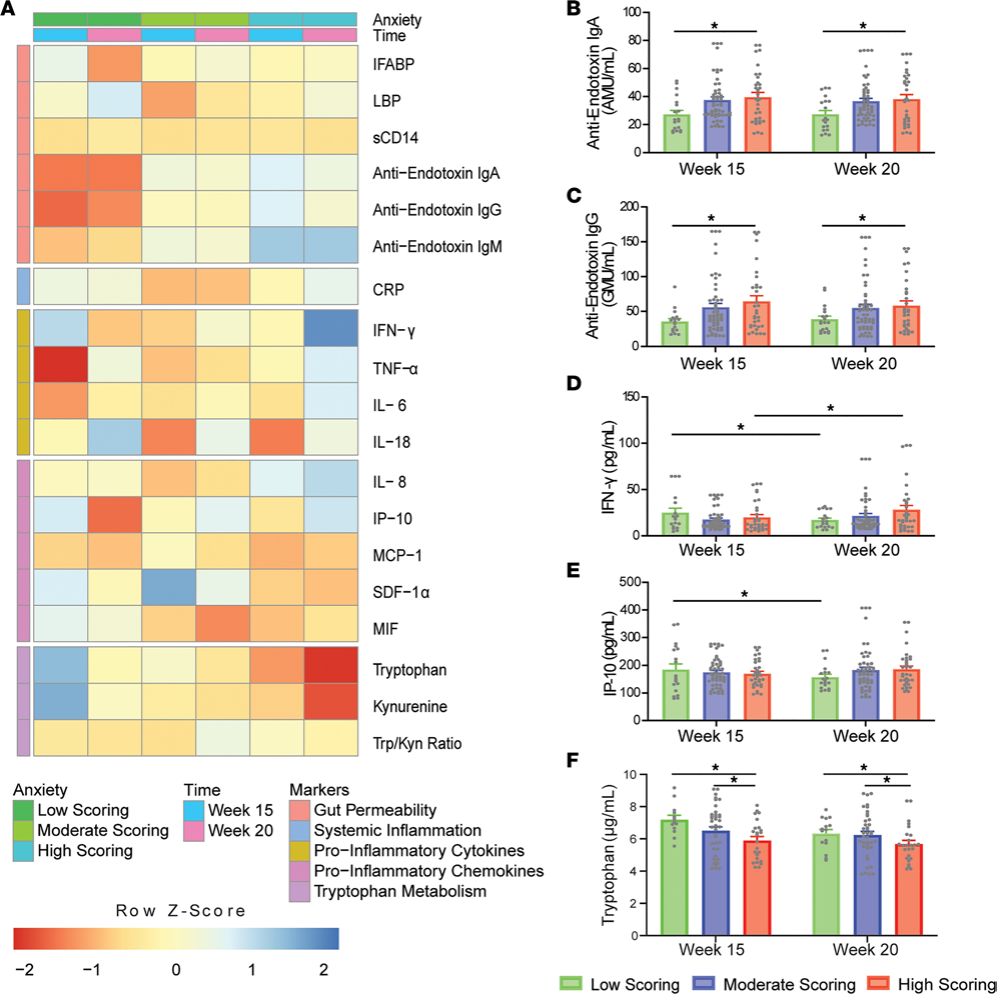
Differences in STAI-scoring groups across gestational time points in the IBS cohort (*N* = 19/53/33 from low/moderate/high). (**A**) Heatmap of normalized biomarker means across scoring groups and time points. Individual boxes are represented on a scale from red to blue. Darker red boxes represent markers whose concentrations were found to be greater than 1 standard deviation lower than the mean concentration across stress group and time point. Darker blue boxes represent markers whose concentrations were found to be greater than 1 standard deviation higher than the mean concentration across stress group and time point. Lower *z* scores are evident for anti-endotoxin IgA and IgG in the low-scoring group at 15 and 20 weeks, for TNF-α in the low-scoring group at 15 weeks, and for Trp and Kyn in the high-scoring group at 20 weeks. Higher *z* scores are evident for Trp and Kyn in the low-scoring group at 15 weeks and for IFN-γ in the high-scoring group at 20 weeks. Significant differences between STAI-scoring groups are illustrated for (**B**) anti-endotoxin IgA, (**C**) anti-endotoxin IgG, (**D**) IFN-γ, (**E**) IP-10, and (**F**) Trp (2-way ANOVA with Tukey’s HSD). *P < 0.05.

Significant differences in STAI scoring groups across gestational time points are outlined in Figure 5. Anti-endotoxin IgA and IgG levels were observed to be significantly higher in the high-scoring group compared with the low-scoring groups (11.59 ± 4.51 IgA median units (AMU)/mL, *P* = 0.031; 26.76 ± 11.11 GMU/mL, *P* = 0.047; respectively). Anti-endotoxin IgA differences between scoring groups were in line with the positive linear association the biomarker exhibited with STAI scores in both simple and adjusted models. A positive association also existed between circulating IgG levels and STAI scores, although this association was no longer significant in the adjusted model. Conversely, Trp levels were found to be significantly decreased in the high-scoring group compared with moderate- (–707.88 ± 289.38 ng/mL, *P* = 0.044) and low-scoring groups (–1111.14 ± 380.08 ng/mL, *P* = 0.013). In addition, both IFN-γ and IP-10 concentrations at 20 weeks’ gestation were noted to be significantly decreased compared with 15 weeks’ gestation (–7.39 ± 3.49 pg/mL, *P* = 0.049; –34.34 ± 14.79 pg/mL, *P* = 0.034; respectively) in low-scoring participants. In contrast, IFN-γ levels were elevated at 20 weeks’ gestation compared with the first visit (8.20 ± 4.0 pg/mL, *P* = 0.049) in the high-scoring group.

#### Associations between maternal depression and biomarker levels in the IBS cohort.

**Figure 6:**
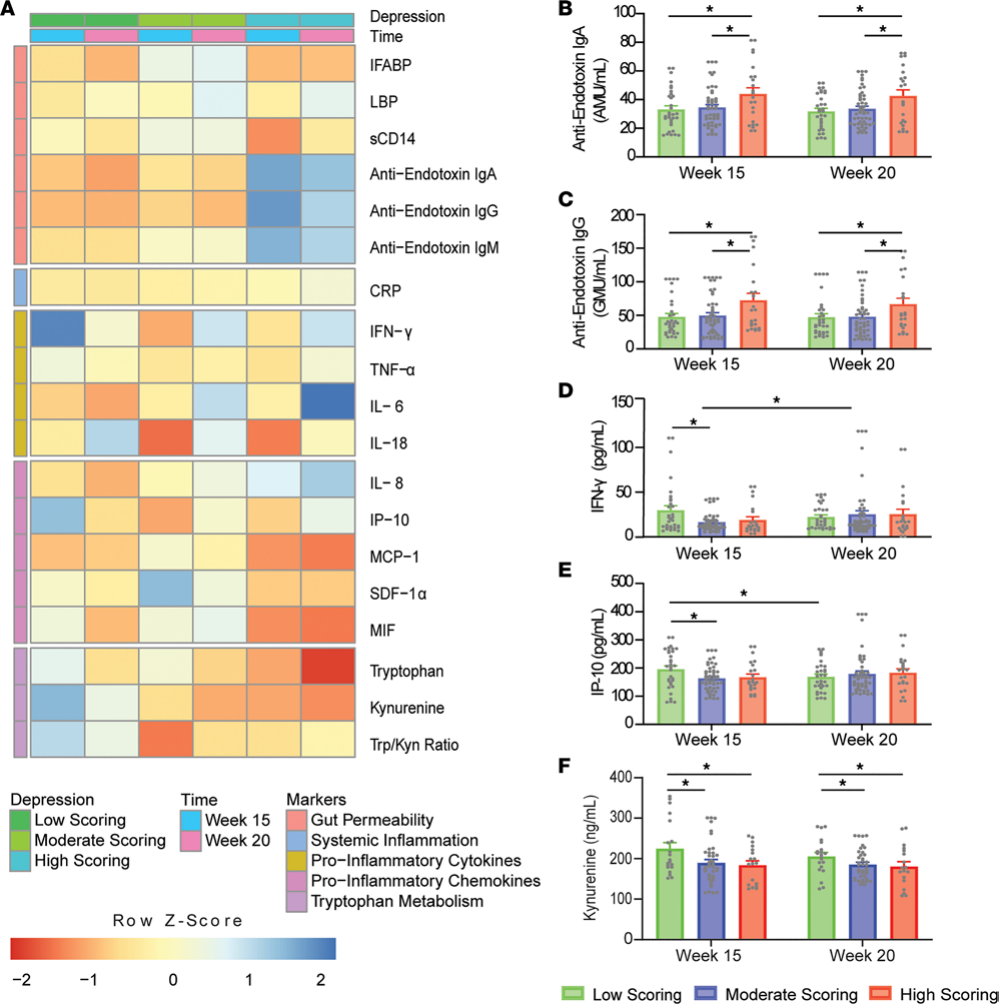
Differences in EPDS-scoring groups across gestational time points in the IBS cohort (*N* = 32/50/23 from low/moderate/high). (**A**) Heatmap of normalized biomarker means across scoring groups and time points. Individual boxes are represented on a scale from red to blue. Darker red boxes represent markers whose concentrations were found to be greater than 1 standard deviation lower than the mean concentration across stress group and time point. Darker blue boxes represent markers whose concentrations were found to be greater than 1 standard deviation higher than the mean concentration across stress group and time point. Lower *z* scores are evident for Trp in the high-scoring group at 20 weeks. Higher *z* scores are evident for IFN-γ in the low-scoring group at 15 weeks; for IL-6 in the high-scoring group at 20 weeks; and for anti-endotoxin IgA, IgG, and IgM in the high-scoring group at 15 and 20 weeks. Significant differences between EPDS-scoring groups are illustrated for (**B**) anti-endotoxin IgA, (**C**) anti-endotoxin IgG, (**D**) IFN-γ, (**E**) IP-10, and (**F**) Kyn (2-way ANOVA with Tukey’s HSD). *P < 0.05.

Significant differences in EPDS scoring groups across gestational time points are outlined in Figure 6. Anti-endotoxin IgA and IgG levels were found to be significantly higher in the high-scoring group compared with moderate (9.22 ± 3.72 AMU/mL, *P* = 0.039; 20.74 ± 8.34 GMU/mL, *P* = 0.038; respectively) and low-scoring groups (10.87 ± 4.04 AMU/mL, *P* = 0.022; 22.13 ± 9.05 GMU/mL, *P* = 0.042; respectively). Differences between scoring groups were in line with a positive linear association exhibited with EPDS scores in the case of anti-endotoxin IgA, but not IgG, for both simple and adjusted models. Conversely, Kyn concentrations were found to be significantly higher in the in the low-scoring group compared with moderate- (34.2 ± 12.13 ng/mL, *P* = 0.017) and high-scoring groups (40.89 ± 14.13 ng/mL, *P* = 0.014). These differences between scoring groups were in line with a negative linear association exhibited with EPDS scores for the simple but not the adjusted model. Additionally, both IFN-γ and IP-10 concentrations were found to be significantly higher in the low-scoring group compared with moderate scores (12.86 ± 4.21 pg/mL, *P* = 0.008; 32.52 ± 12.63 μg/mL, *P* = 0.031; respectively) for measurements taken at 15 weeks’ gestation. However, IFN-γ levels at 20 weeks’ gestation were noted to be significantly elevated compared with 15 weeks’ gestation (8.39 ± 3.97 pg/mL, *P* = 0.040) in moderate-scoring participants, while in the low-scoring group IP-10 levels decreased at 20 weeks’ gestation compared with the first visit (–26.37 ± 8.34 pg/mL, *P* = 0.004).

### Differences in biomarkers between healthy and IBS cohorts

Biomarker concentrations between cohorts across gestational time points are outlined in Supplemental Table 3. Cohort-independent effects revealing significant increases for measurements taken at 20 weeks’ gestation compared with 15 weeks’ gestation were noted for LBP as the mean differences (0.97 ± 0.22 μg/mL, *P* = 0.000), TNF-α (0.07 ± 0.03 pg/mL, *P* = 0.034), IL-6 (0.06 ± 0.03 pg/mL, *P* = 0.017), and IL-18 (71.09 ± 7.26 pg/mL, *P* = 0.000). Conversely, week 20 measurements were observed to be significantly decreased compared with 15 weeks’ gestation for anti-endotoxin IgG (–2.51 ± 0.91 GMU/mL, *P* = 0.006), MCP-1 (-2.49 ± 1.23 pg/mL, *P* = 0.043), and SDF-1α (-38.86 ± 10.78 pg/mL, *P* = 0.001), irrespective of cohort. Cohort-specific effects of time were observed, where levels at 20 weeks’ gestation were found to be significantly decreased for IL-8 (–0.23 ± 0.07 pg/mL, *P* = 0.001) and Trp (–460.22 ± 1477.57 ng/mL, *P* = 0.008) compared with 15 weeks’ gestation in healthy and IBS participants, respectively. For measurements taken at 20 weeks’ gestation, between group effects observed include significant increases in IFN-γ (5.00 ± 2.24 pg/mL, *P* = 0.034) as well as significant decreases in both Trp (–556.52 ± 255.57 ng/mL, *P* = 0.031) and Kyn (–18.29 ± 8.27 ng/mL, *P* = 0.028) in the IBS cohort compared with the healthy cohort. Significant within or between cohort effects were not observed for IFABP, sCD14, anti-endotoxin IgA, anti-endotoxin IgM, CRP, IP-10, MIF, or Trp/Kyn ratio.

## Discussion

### Biological associations with stress during healthy pregnancy.

The present study identifies several molecular candidate markers that exhibit the potential to discern expectant mothers’ level of active stress during the first 2 trimesters of pregnancy in healthy individuals and patients with IBS. In the healthy cohort circulating levels of GI permeability markers sCD14 and LBP were found to be elevated in participants reporting high levels of perceived social stress and depression compared with those reporting low levels. LBP concentrations also exhibited a positive linear relationship with participants’ reported level of anxiety, even after adjusting for common demographic and lifestyle confounders. Additionally, LBP levels were only found to increase across trimester visits in individuals reporting the highest levels of anxiety. A strong case is presented for the systemic inflammatory marker CRP, whose high concentrations distinguished those reporting the highest levels of anxiety and depression from all others. Further support is derived from CRP’s positive linear relationship with anxiety scores, even in adjusted models. Another notable potential biomarker identified in healthy participants is TNF-α, whose circulating concentrations were found to be elevated in participants reporting the highest levels of perceived social stress compared with those reporting low levels.

Observed concentrations of sCD14 and LBP in the healthy cohort were in line with reported findings that GI integrity is undermined by high levels of psychological distress (16). It comes as no surprise that such consistent changes are observed for this biomarker in the context of active stress exposure. Indeed, both play a key role in initiating the innate immune response to the presence of gram-negative bacteria, working in tangent to present bacterial LPS to its signaling receptor complex, MD-2/Toll-like receptor 4 in monocytes (28). Notably, LBP concentrations are reported to fall between 5 and 15 μg/mL in healthy populations (29). Corresponding levels are observed in the present study for the healthy cohort with the prominent exception of participants falling within the high-scoring categories of all reported stress measures (range of means, 16.16–20.10 μg/mL). Modest elevations in LBP levels, equivalent to those presented here in response to prenatal stress, have previously been reported to exist under conditions of chronic inflammation, such as obesity (30) and nonalcoholic fatty liver disease (31). Indeed, these elevations were found to correspond with an upregulated expression of proinflammatory cytokines in these studies. A notable divergence in the stress-mediated effects on sCD14 levels manifested for measures of anxiety, where moderate scores exhibited significantly lower concentrations than the lowest (*P* = 0.027) or highest (*P* = 0.025) reported levels. There is precedent within the literature for positive effects being elicited by mild to moderate levels of maternal stress, particularly in the form of anxiety (32).

The present study also shows that noted associations between elevated CRP levels and increased risk for psychological distress and depression among the general population (33) hold under conditions of pregnancy. Indeed, CRP was found to be the most robust indicator of a shift toward a proinflammatory phenotype in response to prenatal stress among healthy participants. Notably, this acute-phase reactant has been reported to reliably signify the presence of inflammation, even when interpretation from other proinflammatory markers remains ambiguous (34). Corresponding findings in relation to TNF-α under conditions of perceived social stress have notable implications for the cytokine’s potential role as a marker of stress levels, particularly given the role it plays in regulating Trp metabolism via effects on IDO (35). Previously, equivocal results dependent upon the stress stimulus under investigation have been reported regarding the effects of psychological distress on serum TNF-α levels (36). More recently, prenatal stress has been noted to potentiate the effects of a proinflammatory diet on maternal TNF-α concentrations (37). An additional, somewhat paradoxical discovery from the present study was that plasma levels of the proinflammatory chemokine IL-8 were found to be attenuated in participants reporting the highest levels of perceived social stress and depression. Interestingly, this finding concurs with recently reported observations in vitro where cortisol exposure was shown to decrease levels of IL-8 secretion from female peripheral blood mononuclear cells (38). Other inflammatory markers were not found to be associated with perceived stress, anxiety, or depression. In agreement Bränn et al. (39) examined the association between the expression of 74 inflammation-related genes late in pregnancy and the subsequent onset of postpartum depression and failed to find any significant association for inflammatory markers examined in the present study, including IL-6, IP-10, MCP-1, and IL-18. The absence of any significant effect for IL-6 in particular further compounds the conflicting findings in the literature (40), agreeing with the lack of any correlation with depression scores observed by Blackmore et al. (41) at either 18 or 32 weeks’ gestation, but contrasting with reports of positive associations with depressive symptoms in the second trimester (42) as well as trait anxiety and depression scales late in pregnancy (43).

Here we note increases in tryptophan and kynurenine from 15 to 20 weeks’ gestation in the healthy pregnant women. We also noted no association between inflammatory factors and tryptophan in the healthy cohort.

### Biological associations with stress confounded by IBS.

A different picture presents itself in the case of the IBS cohort, where neither sCD14 nor LBP was found to be capable of distinguishing between incremental groupings for any of the stress measures reported in the present study. Instead, robust indications of stress-induced changes on gut permeability are provided by the anti-endotoxin antibodies IgG, whose circulating levels were found to be discernibly elevated at the highest reported levels of all stress measures, and IgA, which exhibited similar findings for reported levels of anxiety and depression. Positive linear associations were also noted between confounder-adjusted concentrations of anti-endotoxin IgA and participants’ self-reported levels of anxiety and depression. Promising findings in relation to proinflammatory markers in the healthy cohort were also not found to be replicated in the IBS cohort. These obvious differences with regard to the association of stress and markers of gut permeability between IBS and non-IBS women have not been shown before (44). While we know the microbiome and gut mucosal inflammation change during a normal healthy pregnancy, we do not know what happens with respect to permeability of the gut wall. Here we see that having IBS leads to a very different biological signature in the plasma potentially due to the already predisposed gut wall, microbiome, and stress system. While we had assumed mothers with IBS would respond to a greater degree to higher stress, anxiety, and depression scores, we did not expect this very different profile. This study indicates the significance of the gut-associated changes in IBS and the need for specific screening and perhaps different intervention strategies.

Equivalent findings were not identified in relation to either sCD14 or LBP under conditions of IBS, a disorder traditionally posited to be characterized by an increased GI response to stress (25). It is plausible that dysregulation of immune signaling pathways under conditions of IBS pathophysiology conceal any potential effects of stress-induced enhancements in gut permeability elicited by these mediators of the innate immune response. Supporting this assertion, evidence has recently emerged establishing several autoimmune diseases, for which dysregulated signaling between immune cells is a hallmark feature (45), as risk factors for IBS independent of the presence of psychological distress (46). Indeed, serum concentrations of LBP and sCD14 have been demonstrated to decrease or remain unchanged under conditions of autoimmunity, such as type 1 diabetes mellitus, despite the presence of elevated levels of LPS (47). Under such conditions the present study finds IgA and IgG antibodies targeted against LPS reliably serve as markers of endotoxin exposure, signifying changes in gut permeability under high levels of self-reported stress. Interestingly, a similar role for these antibodies has previously been reported in patients with major depressive disorder (48) where serum IgM and IgA against LPS were significantly elevated in such cases.

Here we note potential biomarkers for healthy pregnant women during active stress that are indicative of increased GI permeability whereas having the stress-related GI disorder, IBS, is a major confounder to this biomarker panel.

We do not show any evidence for increased metabolism along the Kyn pathway as previously reported in IBS in the nonpregnant state (49). This is not entirely unexpected because we did not note a marked inflammatory phenotype in our IBS cohort. We did see increased production of certain inflammatory markers in the IBS group with higher anxiety and depression scores, but there was not a clear association between inflammation and Trp metabolism; hence, other factors are clearly involved. Lower levels of Trp were present in the women with higher anxiety scores, which has been seen previously (50). A reduction in Kyn was also seen in those with higher depression scores. These differences were observed in the IBS cohort only. This is comparative to a previous study showing an association between lower Kyn levels and depression during pregnancy (51).

### Limitations.

As a consequence of utilizing samples from a pregnancy biobank, there are several limitations associated with the present study. First, although several validated self-reported measures of stress were utilized, no physiological assessment was implemented. Indeed, developmental associations with physiological measures of stress, such as salivary cortisol, have previously been shown to be distinct from self-reported assessments (52), and their investigation is warranted in future studies. Second, in contrast to other investigations into the developmental effects of prenatal stress, which have utilized both generalized and pregnancy-specific assessments of anxiety (52, 53), only a generalized form of anxiety is reported on by the present study. This is of relevance considering that pregnancy-related anxiety measures have previously been demonstrated to be a superior indicator of certain developmental effects over broader assessments of state anxiety (53). Furthermore, measurements were only taken at 2 time points, 5 weeks apart from each other, with no measurements taken in the third trimester of pregnancy. Given that developmental consequences have been associated with the onset of prenatal stress late in pregnancy (54, 55), investigations focusing on these parameters at later time points are justified. Also, given all but 5 subjects included in this study were White, investigation of stress-associated changes in the circulating biomarkers presented here is warranted in other ethnic groups. It should be noted that cases of IBS among participants were self-reported, and as such the possibility cannot be discounted that results may have varied compared with a clinically diagnosed population suffering from functional GI disorders. Finally, given the exploratory nature of this study, associations between individual biomarkers and stress scores are presented on their own merits without adjustment for comparisons made for the other biomarkers investigated. Accordingly, significant associations between stress indicators and circulating biomarkers noted here require validation in future studies.

### Conclusions.

In conclusion, the present study identifies 2 circulating markers of GI permeability (LBP and sCD14) as well as 2 inflammatory markers (CRP and TNF-α) capable of differentiating low and high levels of prenatal maternal stress during otherwise healthy pregnancy. Significantly, although these distinctions were not found to hold under conditions of IBS, endotoxin core antibodies (IgA and IgG) were found to serve as reliable indicators of GI permeability in such cases. Taken together, these biomarkers demonstrate the potential to form the core of a biological signature that could serve as an early warning indicator of high active levels of prenatal maternal stress in women who do not report having IBS. Clinical application of such a signature could inform suitable intervention strategies aimed at counteracting the effects of prenatal maternal stress either via stress reduction techniques or microbiota-targeted nutritional approaches, but it should be noted that having IBS is a major confounder to the validity of these markers. Follow-up studies investigating whether these markers demonstrate any appreciable associations with developmental consequences for the offspring are indicated.

## Methods

### Study cohort.

The Screening for Pregnancy Endpoints (SCOPE) study (56) was a collaborative project that established a unique international pregnancy biobank toward the identification of biomarkers that could be used to predict adverse pregnancy outcomes. The present study consisted of a subset (*n* = 209) of healthy nulliparous women with singleton pregnancies recruited to the Cork cohort of the SCOPE study (*n* = 1774) between November 2004 and January 2011. Women were excluded if underlying medical conditions indicated a high risk of preeclampsia, spontaneous preterm birth, or delivering a small for gestational age infant. Enrolled subjects underwent assessment by a SCOPE research midwife at 15 ± 1 (visit 1) and 20 ± 1 (visit 2) weeks’ gestation. Demographic and clinical characteristics were obtained from subjects during the first visit, including the self-reported presence of IBS, defined as a combination of frequent diarrhea and/or constipation accompanied by abdominal pain and sensation of bloating. Using this classification, all women with IBS and no exclusion criteria (*n* = 105) were included in the study together with an equivalent number of healthy women selected randomly from women with no exclusion criteria and no IBS (*n* = 104). Subject demographics, together with lifestyle characteristics collected at both visits, are presented in Supplemental Table 2. Heparinized blood samples were also collected before 12 pm on the morning of each visit from which plasma was extracted for long-term storage in the SCOPE pregnancy biobank.

### Assessment of maternal psychological status.

Subjects completed a number of clinically validated questionnaires at each visit in order to gauge prenatal levels of stress, anxiety, and depression. The PSS (57) was used to evaluate the degree to which subjects perceived more generalized forms of stress in the month before assessment by appraising their feelings as to how they were able to handle daily hassles, how often they felt nervous and stressed, and how often they felt things were going well. The short form of the state scale of the Spielberger STAI (58) was used to assess the degree to which subjects experienced anxiety-related symptoms or emotions at the time of assessment. Finally, the EPDS (59) was used to determine the presence of depressive symptoms in subjects in the week prior to assessment.

**Figure 7:**
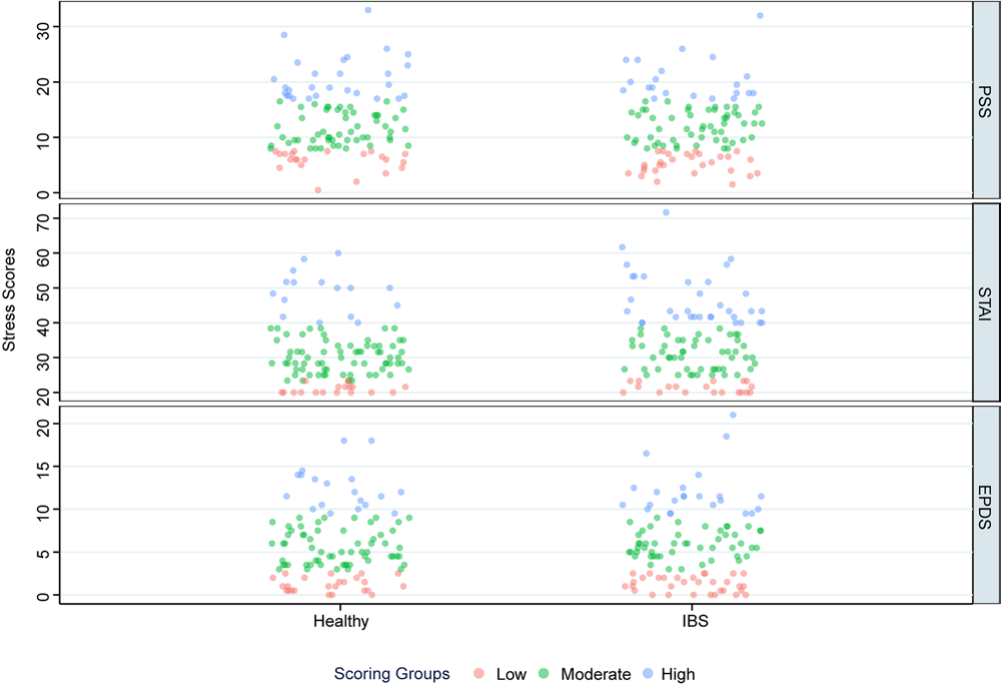
Stratification of perceived stress, anxiety, and depression measures into low-scoring (PSS ≤ 7.5; *N* = 22 and 29 | STAI ≤ 23.3; *N* = 18 and 19 | EPDS ≤ 2.5; *N* = 24 and 32), moderate-scoring (PSS > 7.5 and < 17; *N* = 54 and 55 | STAI > 23.3 and < 40; *N* = 70 and 53 | EPDS > 2.5 and < 9.5; *N* = 61 and 50), and high-scoring groups (PSS ≥ 17; *N* = 28 and 21 | STAI ≥ 40; *N* = 16 and 33 | EPDS ≥ 9.5; *N* = 19 and 23) for healthy and IBS cohorts, respectively.

Lower quartile (PSS — 7.5; STAI — 23.3; EPDS — 2.5) and upper quartile scores (PSS — 17; STAI — 40; EPDS — 9.5) of time point averages were derived from the complete Cork cohort of the SCOPE study and used as cutoffs to define low- (<25th percentile), moderate- (25th to <75th percentile), and high- (≥75th percentile) scoring groups for each of the psychological evaluations. Mean scores across both visits were subsequently used to stratify subjects from the present healthy and IBS cohorts into appropriate scoring groups, as outlined in Figure 7.

A justification for the inclusion of each marker as it relates to GI permeability, systemic inflammation, and Trp metabolism is presented in Supplemental Table 1.

### Measurement of gut permeability markers.

Levels of circulating IFABP and sCD14 (Quantikine Immunoassays, R&D Systems, Bio-Techne), LBP (Hycult Biotech), and anti-endotoxin core antibodies (EndoCAb IgA IgG IgM, Hycult Biotech) were determined using commercially available quantitative ELISAs. For each assay, samples were analyzed in duplicate according to the manufacturers’ instructions with absorbances measured at 450 nm on a Synergy HT BioTek plate reader (Mason Technology). Results were calculated on a 4-parameter logistics curve generated using Gen5 BioTek Microplate Data Collection and Analysis software (Mason Technology). Inter- and intra-assay coefficients of variation for each assay are presented here respectively as follows: IFABP (5.8% and 5.5%), sCD14 (2.5% and 5.8%), LBP (5.1% and 2.7%), anti-endotoxin IgA (5.9% and 9.1%), anti-endotoxin IgG (8.8% and 9.0%), and anti-endotoxin IgM (5.2% and 9.9%).

### Measurement of proinflammatory markers.

Serum concentrations of 4 cytokines (IFN-γ, TNF-α, IL-6, IL-18) and 5 chemokines (IL-8, IP-10, MCP-1, SDF-1α, MIF) were determined using the Meso Scale U-PLEX platform (Meso Scale Diagnostics). This customized multiplex biomarker kit is a high-sensitivity electrochemiluminescence (ECL) immunoassay. Circulating levels of CRP were also assessed by means of ECL immunoassay using the V-PLEX Human CRP Kit (Meso Scale Diagnostics). For each assay, samples were analyzed in duplicate according to the manufacturers’ instructions with ECL measured on a QuickPlex SQ 120 multiplex imager (Meso Scale Diagnostics). Concentrations were calculated from a standard curve calculated using a 4-parameter logistic fit using Workbench 4.0 software (Meso Scale Diagnostics). The inter- and intra-assay coefficients of variation are presented here respectively as follows: IFN-γ (8.7% and 9.5%), TNF-α (6% and 10.3%), IL-6 (6.4% and 9.8%), IL-18 (5.6% and 4.8%), IL-8 (5.4% and 7.2%), IP-10 (6.6% and 6.1%), MCP-1 (6.6% and 6.2%), SDF-1α (9.1% and 8.6%), MIF (12.4% and 10.9%), and CRP (1.5% and 1.8%).

### Measurement of Trp metabolites.

To determine the levels of Trp and Kyn, 198 μL plasma was spiked with internal standard (2 μL) (3-Nitro l-tyrosine) before being deproteinized by the addition of 20 μL of 4 M perchloric acid. Samples were centrifuged at 20,000*g* on Hettich Mikro 22R centrifuge (AGB) for 15 minutes at 4°C and 100 μL of supernatant transferred to an HPLC vial for analysis. Stock solutions of each standard were prepared in HPLC-grade water. Working dilutions were prepared from the stock standards, aliquoted in suitable vials, and stored at –80°C until required for analysis, at which point 20 μL of 4 M perchloric acid was also added and vortexed. Then 20 μL of standards and sample supernatants were vortexed and 20 μL of the supernatant was injected onto the HPLC system (consisting of a CBM-20A system controller, a UV-Vis SPD-10A detector for Kyn, a fluorescence RF-20A detector for Trp, an LC-20AD pump, a CTO-20AC column oven at 30°C, a SIL-20AC HT autosampler, and a Prominence DGU-205R degasser). All samples were injected onto a reverse phase Luna 3 μm C18(2) 100 Å size LC column 150 × 2 mm (Phenomenex), which was protected by Krudkatcher disposable pre-column filters (Phenomenex) and SecurityGuard cartridges (Phenomenex). The mobile phase consisted of 50 mM acetic acid and 100 mM zinc acetate with 3% (*v/v*) acetonitrile and was filtered through MilliporeSigma 0.45 μm HV Durapore membrane filters (AGB) and vacuum degassed prior to use. Compounds were eluted isocratically over a 30-minute run time at a flow rate of 0.3 mL/min after a 20 μL injection. The columns were maintained at a temperature of 30°C, and samples/standards were kept a 4°C in the cooled autoinjector prior to injection. The fluorescence detector was set at an excitation wavelength of 254 nm and an emission wavelength of 404 nm. The UV detector was set at 330 nm. l-Tryptophan and its metabolite kynurenine were identified by their characteristic retention times as determined by injection standards, which were run at regular intervals during the sample analysis. Chromatograms were analyzed using the LabSolutions software (Shimadzu) and concentrations determined using analyte/internal standard peak height ratios. Results were expressed as ng/mL of supernatant.

### Statistics.

All data are presented as mean ± SEM, unless otherwise indicated. A 2-way repeated measures ANOVA was run to elucidate differences between healthy and IBS cohorts across gestational time points on biomarker concentrations using SPSS, version 25.0 (SPSS Inc.). An equivalent analysis was also performed for each cohort individually to determine the effect of different scoring groups for each stress measure over time on each biomarker, with Tukey’s HSD used for post hoc analysis between individual scoring groups. Perceived stress, anxiety, and depression scores were also investigated as continuous response variables against each biomarker in multiple linear regression models carried out in R. Biomarker coefficients adjusted for age, BMI, socioeconomic status, smoking status, and alcohol intake prior to participation in the study are reported. Statistical significance was accepted at the *P* < 0.05 level of confidence for all models.

### Study approval.

The research described received approval from the Clinical Research Ethics Committee of the Cork Teaching Hospitals (protocol number: APC1004; approval number: APC-D-14). Informed consent was obtained from all participants, who were free to withdraw from the study at any time.

## Author contributions

JMK, JMC, SOD, JB, and ASK were involved in conducting experiments, acquiring data, and analyzing data. ASK, FPM, JFC, TGD, GC, and SOM were involved in designing the study and acquired the funding. JMK, ASK, FPM, GC, JFC, and SOM were involved in writing the manuscript. LCK provided the samples for the analysis.

## Supplementary Material

Supplemental data

## Version 1 12/10/2020

In-Press Preview

## Version 2 01/25/2021

Electronic publication

## References

[1] Lilliecreutz C (2016). Effect of maternal stress during pregnancy on the risk for preterm birth. BMC Pregnancy Childbirth.

[2] Khashan AS (2014). Second-trimester maternal distress increases the risk of small for gestational age. Psychol Med.

[3] Maher GM (2017). Hypertensive disorders of pregnancy and risk of neurodevelopmental disorders in the offspring: a systematic review and meta-analysis protocol. BMJ Open.

[4] Glover V (2019). Is the association between maternal stress during pregnancy and the child’s depression partly causal, and what should we do about it?. Acta Psychiatr Scand.

[5] Chan JC (2018). Parental advisory: maternal and paternal stress can impact offspring neurodevelopment. Biol Psychiatry.

[6] Gur TL (2019). Prenatal stress disrupts social behavior, cortical neurobiology and commensal microbes in adult male offspring. Behav Brain Res.

[7] Gur TL (2017). Prenatal stress affects placental cytokines and neurotrophins, commensal microbes, and anxiety-like behavior in adult female offspring. Brain Behav Immun.

[8] Hartman S (2020). Does prenatal stress amplify effects of postnatal maternal depressive and anxiety symptoms on child problem behavior?. Dev Psychol.

[9] Laplante DP (2004). Stress during pregnancy affects general intellectual and language functioning in human toddlers. Pediatr Res.

[10] Van den Bergh BRH (2017). Prenatal developmental origins of behavior and mental health: the influence of maternal stress in pregnancy. Neurosci Biobehav Rev.

[11] Weinstock M (2008). The long-term behavioural consequences of prenatal stress. Neurosci Biobehav Rev.

[12] Patterson PH (2009). Immune involvement in schizophrenia and autism: etiology, pathology and animal models. Behav Brain Res.

[13] Cryan JF (2019). The microbiota-gut-brain axis. Physiol Rev.

[14] Power SE (2014). Intestinal microbiota, diet and health. Br J Nutr.

[15] Vaarala O (2008). The “perfect storm” for type 1 diabetes: the complex interplay between intestinal microbiota, gut permeability, and mucosal immunity. Diabetes.

[16] Vanuytsel T (2014). Psychological stress and corticotropin-releasing hormone increase intestinal permeability in humans by a mast cell-dependent mechanism. Gut.

[17] Kelly JR (2015). Breaking down the barriers: the gut microbiome, intestinal permeability and stress-related psychiatric disorders. Front Cell Neurosci.

[18] Jiang NM (2018). The impact of systemic inflammation on neurodevelopment. Trends Mol Med.

[19] Notarangelo FM, Pocivavsek A (2017). Elevated kynurenine pathway metabolism during neurodevelopment: implications for brain and behavior. Neuropharmacology.

[20] O’Mahony SM (2015). Serotonin, tryptophan metabolism and the brain-gut-microbiome axis. Behav Brain Res.

[21] Black CJ, Ford AC (2020). Global burden of irritable bowel syndrome: trends, predictions and risk factors. Nat Rev Gastroenterol Hepatol.

[22] Clarke G (2009). Irritable bowel syndrome: towards biomarker identification. Trends Mol Med.

[23] Enck P (2016). Irritable bowel syndrome. Nat Rev Dis Primers.

[24] Kennedy PJ (2014). Irritable bowel syndrome: a microbiome-gut-brain axis disorder?. World J Gastroenterol.

[25] Qin HY (2014). Impact of psychological stress on irritable bowel syndrome. World J Gastroenterol.

[26] Black CJ (2020). Anxiety-related factors associated with symptom severity in irritable bowel syndrome. Neurogastroenterol Motil.

[27] Fond G (2014). Anxiety and depression comorbidities in irritable bowel syndrome (IBS): a systematic review and meta-analysis. Eur Arch Psychiatry Clin Neurosci.

[28] Kitchens RL, Thompson PA (2005). Modulatory effects of sCD14 and LBP on LPS-host cell interactions. J Endotoxin Res.

[29] Gallay P (1994). Lipopolysaccharide (LPS)-binding protein in human serum determines the tumor necrosis factor response of monocytes to LPS. J Infect Dis.

[30] Gonzalez-Quintela A (2013). Determinants of serum concentrations of lipopolysaccharide-binding protein (LBP) in the adult population: the role of obesity. PLoS One.

[31] Ruiz AG (2007). Lipopolysaccharide-binding protein plasma levels and liver TNF-alpha gene expression in obese patients: evidence for the potential role of endotoxin in the pathogenesis of non-alcoholic steatohepatitis. Obes Surg.

[32] DiPietro JA (2006). Maternal psychological distress during pregnancy in relation to child development at age two. Child Dev.

[33] Wium-Andersen MK (2013). Elevated C-reactive protein levels, psychological distress, and depression in 73, 131 individuals. JAMA Psychiatry.

[34] Karadag F (2008). The value of C-reactive protein as a marker of systemic inflammation in stable chronic obstructive pulmonary disease. Eur J Intern Med.

[35] Robinson CM (2005). The role of IFN-gamma and TNF-alpha-responsive regulatory elements in the synergistic induction of indoleamine dioxygenase. J Interferon Cytokine Res.

[36] Chandrashekara S (2007). Effects of anxiety on TNF-alpha levels during psychological stress. J Psychosom Res.

[37] Lindsay KL (2018). Maternal stress potentiates the effect of an inflammatory diet in pregnancy on maternal concentrations of tumor necrosis factor alpha. Nutrients.

[38] Da Pozzo E (2018). Cytokine secretion responsiveness of lymphomonocytes following cortiso l cell exposure: sex differences. PLoS One.

[39] Bränn E (2017). Inflammatory markers in late pregnancy in association with postpartum depression-a nested case-control study. Psychoneuroendocrinology.

[40] Osborne LM, Monk C (2013). Perinatal depression--the fourth inflammatory morbidity of pregnancy?: Theory and literature review. Psychoneuroendocrinology.

[41] Blackmore ER (2011). Psychiatric symptoms and proinflammatory cytokines in pregnancy. Psychosom Med.

[42] Sherer ML (2018). The psychoneuroimmunology of pregnancy. Front Neuroendocrinol.

[43] Maes M (2000). Immune activation in the early puerperium is related to postpartum anxiety and depressive symptoms. Psychoneuroendocrinology.

[44] Edwards SM (2017). The maternal gut microbiome during pregnancy. MCN Am J Matern Child Nurs.

[45] Arakelyan A (2017). Autoimmunity and autoinflammation: a systems view on signaling pathway dysregulation profiles. PLoS One.

[46] Koloski N (2019). Population based study: atopy and autoimmune diseases are associated with functional dyspepsia and irritable bowel syndrome, independent of psychological distress. Aliment Pharmacol Ther.

[47] Aravindhan V (2015). Chronic endotoxemia in subjects with type-1 diabetes is seen much before the onset of microvascular complications. PLoS One.

[48] Maes M (2008). The gut-brain barrier in major depression: intestinal mucosal dysfunction with an increased translocation of LPS from gram negative enterobacteria (leaky gut) plays a role in the inflammatory pathophysiology of depression. Neuro Endocrinol Lett.

[49] Clarke G (2009). Tryptophan degradation in irritable bowel syndrome: evidence of indoleamine 2,3-dioxygenase activation in a male cohort. BMC Gastroenterol.

[50] Songtachalert T (2018). Anxiety disorders: sex differences in serotonin and tryptophan metabolism. Curr Top Med Chem.

[51] Nazzari S (2020). Prenatal IL-6 levels and activation of the tryptophan to kynurenine pathway are associated with depressive but not anxiety symptoms across the perinatal and the post-partum period in a low-risk sample. Brain Behav Immun.

[52] Bennet SM (2016). Global cytokine profiles and association with clinical characteristics in patients with irritable bowel syndrome. Am J Gastroenterol.

[53] Davis EP, Sandman CA (2010). The timing of prenatal exposure to maternal cortisol and psychosocial stress is associated with human infant cognitive development. Child Dev.

[54] Moss KM (2017). A potential psychological mechanism linking disaster-related prenatal maternal stress with child cognitive and motor development at 16 months: the QF2011 Queensland flood study. Dev Psychol.

[55] Simcock G (2017). Infant neurodevelopment is affected by prenatal maternal stress: the QF2011 Queensland flood study. Infancy.

[56] Kenny LC (2014). Early pregnancy prediction of preeclampsia in nulliparous women, combining clinical risk and biomarkers: the Screening for Pregnancy Endpoints (SCOPE) international cohort study. Hypertension.

[57] Cohen S (1983). A global measure of perceived stress. J Health Soc Behav.

[58] Marteau TM, Bekker H (1992). The development of a six-item short-form of the state scale of the Spielberger State-Trait Anxiety Inventory (STAI). Br J Clin Psychol.

[59] Cox JL (1996). Validation of the Edinburgh Postnatal Depression Scale (EPDS) in non-postnatal women. J Affect Disord.

