# Supplementary material for: Identifying a biological signature of prenatal maternal stress
Source: JCI Insight. 2021 Jan 25;6(2):e143007. doi: 10.1172/jci.insight.143007 (PMC7934857; doi:10.1172/jci.insight.143007)
Supplement: Supplemental data [file jciinsight-6-143007-s108.pdf]

Gut Permeability	
**Intestinal Fatty Acid Binding Protein (IFABP)**	Small, water-soluble cytosolic proteins which are easily released into the circulation upon enterocyte membrane integrity loss. Basal levels of have been reported to reflect the physiological turnover rate of enterocytes (60).
**Lipopolysaccharide Binding Protein (LBP)**	An acute phase protein involved in initiation of host defence against Gram-negative bacteria by binding to bacterial lipopolysaccharide (LPS) and presenting it to the cell surface pattern recognition receptors CD14 and TLR4 (61).
**Soluble CD14 (sCD14)**	Released by macrophages upon stimulation with endotoxin and acts as a co-factor, along with LBP, to mediate LPS recognition and initiate an innate immune response (61).
**Endotoxin Core Antibodies (IgA; IgG; IgM)**	EndoCAb assays detect immunoglobulins against the inner core of endotoxin which is highly conserved across the whole range of Gram-negative microbiota (60). Previously shown to be upregulated in cases of major depression indicating an increased translocation of LPS from gram negative enterobacteria into the circulation (48).
Systemic Inflammation	
**C Reactive Protein (CRP)**	Acute phase protein which have been shown to be a valid biomarker of low-grade systemic inflammation (34).
Pro-Inflammatory Cytokines	
**Interferon Gamma (IFN-γ)**	Significantly induced in response to psychological stress (62), IFN-γ is associated with disturbances of serotonergic signalling through activation of IDO, the rate-limiting enzyme for tryptophan degradation (63).
**Tumor Necrosis Factor Alpha (TNF-α)**	Significantly increased in response to psychological stress, TNF-α is involved in activating the HPA axis and causing the release of cortisol (64). Attenuation of TNF-α signalling was shown to prevent placental defects caused by mild maternal immune activation in early pregnancy (65). IFN-γ-induced IDO expression is also potentiated by TNF- α (35).
**Interleukin 6 (IL-6)**	Implicated as a mediating factor in processes leading from maternal inflammation to alterations in fetal brain development (66).
**Interleukin 18 (IL-18)**	Stress induced increases in IL-18 is found to be dependent upon the presence of microbiota derived LPS in the circulation (67). Also found to suppress neuronal survival and differentiation in embryonic neural progenitor culture (68).
Pro-Inflammatory Chemokines	
**Interleukin 8 (IL-8 / CXCL8)**	Postulated biomarker of chronic stress (69), whose concentrations are found to correlate between maternal and neonatal serum (70). Its expression was also found to correlate positively with the severity of preeclampsia (71) and high maternal serum levels were found to be indicative of preterm labour (72).
**Interferon Gamma-induced Protein 10 (IP-10 / CXCL10)**	Highly expressed in response to IFN-γ, with significantly high concentrations observed in patients with preeclampsia (73) as well as mother who deliver pre-term (74).
**Monocyte Chemoattractant Protein 1 (MCP-1 / CCL2)**	Significantly increased in women in response to prolonged psychosocial stress (75) as well as populations suffering from generalised anxiety disorder (76). MCP-1 concentrations in cord blood were found to be associated with intrauterine inflammation, premature birth, and neonatal complications (77).
**Stromal cell-derived Factor 1 (SDF-1 α / CXCL12)**	Implicated in regulating interactions between the immune and nervous systems (78), circulating levels are found to be elevated in populations suffering from depression (79), generalised anxiety disorder (76) and post-traumatic stress disorder (80).
**Macrophage Migration Inhibitory Factor (MIF)**	Exhibits a similar circadian rhythm to plasma cortisol and promotes the expression of a large panel of pro-inflammatory molecules by antagonizing cortisol-mediated pro-inflammatory cytokine suppression (81).
Tryptophan Metabolism	
**Tryptophan (Trp)**	Amino acid whose inflammatory mediated breakdown is associated with enhanced sensitivity to anxiety (82) and depression (83).
**Kynurenine (Kyn)**	Metabolite of tryptophan whose production is implicated in both inflammatory and neurological conditions (84).
**Kynurenine : Tryptophan Ratio (Kyn/Trp)**	A measure of tryptophan degradation along the Kyn pathway which may represent a key mediator of the physiological consequences of altered immunoregulation (49).

Supplementary Table 1. Explanation of how the biological characteristics investigated in the present study serve as molecular indicators of gut permeability, inflammation and tryptophan degradation associated with heightened levels of prenatal stress.

	Healthy (N = 104)	IBS (N = 105)
Age (Years)	30.06±3.99	30.68±4.33
BMI at 15 Week Visit (kg/m2)	24.4±3.97	24.94±3.86
Alcohol Exposure in 1st Trimester (Weeks)	4.97±4.29	4.73±4.72
Alcohol Intake at 20 Week Visit (Units per Week)	0.21±0.52	0.25±0.78
**Ethnicity**		
Caucasian	95.19%	100%
Asian	0.96%	-
Indian	3.85%	-
**Marital Status**		
Partner	93.33%	10.48%
Single	6.67%	89.52%
**Years in Education**		
< 12yrs	0.95%	1.9%
12-13yrs	60.95%	64.76%
>13yrs	38.10%	33.33%
**Employment Status**		
Full Time	84.76%	76.19%
Part Time	10.48%	7.62%
Student	1.9%	-
Homemaker	0.95%	2.86%
Unemployed	1.9%	9.52%
Sickness Beneficiary	-	1.9%
Other	-	1.9%
**Socioeconomic Index (SEI)***		
Low (SEI < 24)	12.50%	17.14%
High (SEI ≥ 24)	87.50%	82.86%
**Type of Maternity Care**		
Public	74.04%	69.52%
Private	25.96%	30.48%
**Cigarettes Smoked (15 Week Visit)**		

None	94.23%	89.52%
1-5 per Day	3.85%	7.62%
6-10 per Day	1.92%	2.86%
**Cigarettes Smoked (20 Week Visit)**		
None	92.31%	88.58%
1-5 per Day	4.81%	5.71%
6-10 per Day	2.88%	5.71%

Supplementary Table 2. Descriptive statistics for demographic and lifestyle characteristics for healthy and IBS cohorts. Continuous variables are presented as mean ± SD. * Maternal Socioeconomic index (SEI) calculated using the New Zealand Socioeconomic Index guide (Galbraith et al., 1996).

		Healthy		IBS	
	Time	Week 15	Week 20	Week 15	Week 20
**Stress Scores**					
PSS		13.9 ± 6.8	11.6 ± 6.8	13.4 ± 6.5	10.2 ± 6.6
STAI		**31.6 ± 10.6***	31.4 ± 9.9	**35.8 ± 12.7***	32.6 ± 11.9
EPDS		6.3 ± 4.7	5.3 ± 4.3	6.8 ± 4.9	4.8 ± 4.5
**Gut Permeability**					
IFABP (pg/mL)		5.9 ± 3.0	5.7 ± 2.5	5.7 ± 2.8	5.6 ± 2.7
LBP (μg/mL)	***	**14.9 ± 5.7**	**15.5 ± 6.3**	**15.1 ± 6.0**	**16.3 ± 6.4**
sCD14 (ng/mL)		920.3 ± 164.7	900.7 ± 167.4	897.5 ± 189.3	900.1 ± 169.1
Anti-Endotoxin IgA (AMU/mL)		35.2 ± 17.1	35.3 ± 17.8	36.3 ± 16.5	35.4 ± 15.6
Anti-Endotoxin IgG (GMU/mL)	**	**53.9 ± 38.3**	**52.4 ± 38.1**	**56.3 ± 40.3**	**52.8 ± 35.1**
Anti-Endotoxin IgM (MMU/mL)		54.5 ± 23.6	55.1 ± 22.0	56.2 ± 25.9	56.2 ± 23.7
**Systemic Inflammation**					
CRP (μg/mL)		5.5 ± 3.8	5.6 ± 3.8	5.8 ± 4.0	5.8 ± 4.0
**Pro-Inflammatory Cytokines**					
IFN-γ (pg/mL)		18.6 ± 13.7	**18.0 ± 11.9***	19.2 ± 14.3	**23.0 ± 20.7***
TNF-α (pg/mL)	*	**0.917 ± 0.914**	**0.965 ± 0.979**	**0.741 ± 0.701**	**0.833 ± 0.812**
IL-6 (pg/mL)	*	**0.564 ± 0.366**	**0.576 ± 0.352**	**0.538 ± 0.381**	**0.646 ± 0.528**
IL-18 (pg/mL)	***	**424.1 ± 147.1**	**481.2 ± 168.7**	**400.9 ± 144.6**	**485.9 ± 191.6**
**Pro-Inflammatory Chemokines**					
IL-8 (pg/mL)		**2.6 ± 0.9****	**2.4 ± 0.7****	2.5 ± 0.9	2.6 ± 1.0
IP-10 (pg/mL)		170.6 ± 58.9	171.6 ± 61.9	174.0 ± 57.2	177.8 ± 66.1
MCP-1 (pg/mL)	*	**118.8 ± 31.1**	**115.1 ± 27.7**	**110.2 ± 28.8**	**108.9 ± 25.5**
SDF-1α (pg/mL)	***	**939.9 ± 220.1**	**900.9 ± 232.4**	**948.6 ± 219.9**	**909.8 ± 217.7**
MIF (ng/mL)		23.2 ± 16.7	22.3 ± 14.6	21.5 ± 16.2	20.7 ± 14.6
**Tryptophan Metabolism**					
Trp (ng/mL)		6523.3±1786.7	**6638.9±1821.4***	**6568.5±1571.9***	**6108.3±1331.7***
Kyn (ng/mL)		197±50.9	**205.3±55.9***	198.3±55.6	**188.4±45.7***
Kyn/Trp		0.031±0.006	0.031±0.006	0.03±0.006	0.031±0.007

Supplementary Table 3. Descriptive statistics are outlined for each stress score and biomarker across gestational time-points for healthy and IBS cohorts. Data is presented as mean ± SD. Effects between cohorts are highlighted in bold. Cohort specific effects across time-points are also highlighted in bold and underlined. Significance levels (* P<0.05; ** P<0.01; *** P<0.001) are indicated in the appropriate columns. Significance is also indicated in the Time column where biomarker levels differ across time-points irrespective of cohort.

	Healthy		IBS	
	Week 15	Week 20	Week 15	Week 20
	β (95% CI)	β (95% CI)	β (95% CI)	β (95% CI)
**Gut Permeability**				
IFABP	0.0001 (-0.0003 – 0.0005)	0.0003 (-0.0001 – 0.0007)	-0.0002 (-0.0006 – 0.0001)	0.0001 (-0.0003 – 0.0004)
	0.0001 (-0.0002 – 0.0005)	0.0002 (-0.0002 – 0.0007)	-0.0001 (-0.0005 – 0.0002)	0 (-0.0004 – 0.0004)
LBP	0.1019 (-0.0901 – 0.2939)	0.1624 (-0.0063 – 0.3311)	-0.0274 (-0.201 – 0.1462)	0.0191 (-0.1443 – 0.1825)
	0.1007 (-0.0879 – 0.2893)	0.1463 (-0.0189 – 0.3114)	-0.026 (-0.201 – 0.149)	-0.0023 (-0.1661 – 0.1615)
sCD14	0.0047 (-0.0019 – 0.0113)	0.004 (-0.0024 – 0.0105)	-0.0019 (-0.0074 – 0.0036)	-0.0016 (-0.0078 – 0.0046)
	0.0031 (-0.0036 – 0.0098)	0.0021 (-0.0045 – 0.0087)	-0.0013 (-0.0067 – 0.004)	-0.0019 (-0.0081 – 0.0042)
Anti-Endotoxin IgA	-0.0094 (-0.0728 – 0.054)	-0.0164 (-0.077 – 0.0442)	0.0476 (-0.015 – 0.1102)	0.051 (-0.0151 – 0.1172)
	-0.0158 (-0.079 – 0.0474)	-0.0101 (-0.071 – 0.0503)	0.0461 (-0.0188 – 0.111)	0.0401 (-0.0258 – 0.106)
Anti-Endotoxin IgG	0.0117 (-0.0164 – 0.0399)	0.0029 (-0.0255 – 0.0313)	**0.0288 (0.0035 – 0.0541)***	**0.0296 (0.0004 – 0.0588)***
	0.0033 (-0.0256 – 0.0322)	0.0044 (-0.024 – 0.0329)	0.0215 (-0.0037 – 0.0467)	0.0224 (-0.0071 – 0.0518)
Anti-Endotoxin IgM	0.0309 (-0.0146 – 0.0764)	0.023 (-0.026 – 0.072)	0.0297 (-0.0102 – 0.0696)	0.0299 (-0.0137 – 0.0736)
	0.024 (-0.0226 – 0.0705)	0.019 (-0.0298 – 0.0677)	0.0235 (-0.0157 – 0.0627)	0.0247 (-0.018 – 0.0675)
**Systemic Inflammation**				
CRP	0.1495 (-0.1345 – 0.4335)	0.1791 (-0.1069 – 0.4652)	-0.0389 (-0.3028 – 0.2251)	0.0817 (-0.1779 – 0.3413)
	0.1325 (-0.1485 – 0.4136)	0.1378 (-0.1499 – 0.4255)	0 (-0.2633 – 0.2634)	0.1287 (-0.1322 – 0.3897)
**Pro-Inflammatory Cytokines**				
IFN-γ	-0.0268 (-0.1056 – 0.052)	-0.0014 (-0.092 – 0.0893)	-0.0394 (-0.1118 – 0.033)	0.0178 (-0.0325 – 0.068)
	-0.0074 (-0.086 – 0.0712)	-0.0014 (-0.0903 – 0.087)	-0.0272 (-0.1016 – 0.0472)	0.0069 (-0.0429 – 0.0568)
TNF-α	0.8678 (-0.3041 – 2.0398)	0.7697 (-0.325 – 1.8644)	-0.0208 (-1.51 – 1.4683)	0.3277 (-0.9558 – 1.6112)
	0.678 (-0.4891 – 1.8452)	0.664 (-0.4226 – 1.7505)	0.1576 (-1.3264 – 1.6415)	0.4399 (-0.8199 – 1.6998)
IL-6	0.0461 (-2.9141 – 3.0063)	0.0767 (-3.0022 – 3.1557)	0.6293 (-2.106 – 3.3647)	1.1606 (-0.8009 – 3.1221)
	-0.0948 (-3.0734 – 2.8838)	0.3091 (-2.8641 – 3.4823)	0.8531 (-1.8962 – 3.6025)	1.0233 (0.9473 – 2.9939)
IL-18	-0.0013 (-0.0087 – 0.006)	0.0011 (-0.0053 – 0.0075)	-0.0055 (-0.0126 – 0.0016)	-0.0026 (-0.008 – 0.0028)
	0 (-0.0077 – 0.0077)	0.0021 (-0.0042 – 0.0084)	-0.0064 (-0.0133 – 0.0005)	-0.003 (-0.0083 – 0.0023)
**Pro-Inflammatory Chemokines**				
IL-8	-0.6673 (-1.8985 – 0.564)	-1.4563 (-3.032 – 0.1192)	0.5453 (-0.6327 – 1.7232)	0.7839 (-0.2466 – 1.8144)
	-0.7488 (-1.9715 – 0.4739)	-1.1998 (-2.756 – 0.3563)	0.5271 (-0.6479 – 1.702)	0.6588 (-0.3784 – 1.696)
IP-10	-0.0077 (-0.0261 – 0.0106)	0.0065 (-0.011 – 0.0239)	-0.0168 (-0.0348 – 0.0011)	0.0035 (-0.0123 – 0.0193)
	-0.0035 (-0.0221 – 0.015)	0.0094 (-0.0079 – 0.0268)	-0.0163 (-0.0345 – 0.002)	0.0021 (-0.0134 – 0.0176)
MCP-1	-0.0304 (-0.0647 – 0.0039)	-0.0293 (-0.0678 – 0.009)	-0.0136 (-0.0497 – 0.0225)	-0.0122 (-0.053 – 0.0286)
	-0.0327 (-0.0666 – 0.0012)	-0.0277 (-0.0664 – 0.011)	-0.015 (-0.051 – 0.0209)	-0.0098 (-0.0497 – 0.03)
SDF-1 α	-0.0032 (-0.008 – 0.0017)	-0.0012 (-0.006 – 0.0034)	-0.001 (-0.0057 – 0.0038)	0.001 (-0.0038 – 0.0058)
	-0.0028 (-0.0077 – 0.0021)	-0.0009 (-0.006 – 0.0039)	-0.0007 (-0.0054 – 0.0041)	0.0022 (-0.0026 – 0.0069)
MIF	0.047 (-0.017 – 0.111)	0.0389 (-0.035 – 0.1128)	-0.0217 (-0.086 – 0.0425)	-0.0178 (-0.0889 – 0.0534)
	0.0335 (-0.0298 – 0.0969)	0.0318 (-0.0406 – 0.1041)	-0.0147 (-0.0779 – 0.0485)	-0.0104 (-0.0797 – 0.0588)
**Tryptophan Metabolism**				
Trp	-0.0005 (-0.0012 – 0.0002)	0.0001 (-0.0006 – 0.0007)	-0.0006 (-0.0014 – 0.0002)	-0.0005 (-0.0014 – 0.0004)
	-0.0006 (-0.0013 – 0.0001)	-0.0001 (-0.0008 – 0.001)	-0.0002 (-0.001 – 0.0006)	-0.0004 (-0.0013 – 0.0005)
Kyn	-0.0097 (-0.0344 – 0.0149)	-0.0007 (-0.023 – 0.0213)	-0.0155 (-0.0372 – 0.0062)	-0.0119 (-0.0388 – 0.015)
	-0.0099 (-0.0359 – 0.0161)	0.0001 (-0.023 – 0.023)	-0.0052 (-0.0276 – 0.0172)	-0.0042 (-0.0318 – 0.0234)
Kyn/Trp	158.7 (-57.6 – 374.9)	49.5 (-184.4 – 283.4)	33.5 (-167.9 – 235.1)	21.7 (-170.2 – 213.6)
	201.5 (-28.6 – 431.6)	173.7 (-93.9 – 441.4)	36.9 (-172.6 – 246.4)	73.8 (-116.4 – 264.1)

Supplementary Table 4. Regression coefficients and confidence intervals are presented for linear models where PSS scores are regressed against biomarker levels both in isolation (rows with clear background) as well as adjusted for

age, BMI, socioeconomic index, smoking status and alcohol intake (rows with shaded background). Significant coefficients are highlighted in bold (* P<0.05; ** P<0.01; *** P<0.001).

	Healthy		IBS	
	**Week 15**	**Week 20**	**Week 15**	**Week 20**
	**β (95% CI)**	**β (95% CI)**	**β (95% CI)**	**β (95% CI)**
**Gut Permeability**				
IFABP	0.0004 (-0.0002 – 0.001)	0.0003 (-0.0003 – 0.001)	-0.0002 (-0.0009 – 0.0005)	0 (-0.0007 – 0.0007)
	0.0004 (-0.0001 – 0.001)	0.0003 (-0.0004 – 0.001)	0 (-0.0007 – 0.0006)	-0.0001 (-0.0008 – 0.0006)
LBP	**0.355 (0.0562 – 0.6537)***	**0.3785 (0.116 – 0.641)****	-0.0512 (-0.3739 – 0.2714)	-0.0011 (-0.3049 – 0.3026)
	**0.3434 (0.0514 – 0.6355)***	**0.3713 (0.114 – 0.628)****	-0.048 (-0.3649 – 0.2689)	-0.0045 (-0.3062 – 0.2971)
sCD14	0.0015 (-0.0092 – 0.0121)	0.0061 (-0.0041 – 0.0164)	-0.003 (-0.0132 – 0.0073)	-0.0028 (-0.0143 – 0.0086)
	-0.0007 (-0.0113 – 0.0099)	0.0046 (-0.0059 – 0.0151)	-0.0025 (-0.0122 – 0.0072)	-0.0025 (-0.0138 – 0.0089)
Anti-Endotoxin IgA	-0.0636 (-0.1635 – 0.0363)	-0.0905 (-0.1851 – 0.004)	**0.1385 (0.0251 – 0.2518)***	**0.1429 (0.0229 – 0.2628)***
	-0.077 (-0.1754 – 0.0214)	-0.0811 (-0.1762 – 0.014)	**0.1562 (0.0423 – 0.2701)****	**0.1212 (0.0021 – 0.2402)***
Anti-Endotoxin IgG	0.0075 (-0.0373 – 0.0523)	-0.012 (-0.0571 – 0.033)	**0.0507 (0.0041 – 0.0973)***	**0.06 (0.0065 – 0.1134)***
	-0.0087 (-0.0542 – 0.0368)	-0.015 (-0.0601 – 0.0306)	0.0345 (-0.0111 – 0.0802)	0.0494 (-0.0042 – 0.1031)
Anti-Endotoxin IgM	-0.0063 (-0.0729 – 0.0666)	-0.0321 (-0.1099 – 0.046)	0.0433 (-0.0304 – 0.117)	0.0515 (-0.0289 – 0.1319)
	-0.0117 (-0.0854 – 0.0619)	-0.0296 (-0.1074 – 0.048)	0.0338 (-0.0371 – 0.1047)	0.0318 (-0.0467 – 0.1103)
**Systemic Inflammation**				
CRP	**0.507 (0.0651 – 0.949)***	**0.5535 (0.1092 – 0.998)***	-0.0364 (-0.5223 – 0.4496)	0.2129 (-0.2639 – 0.6897)
	**0.443 (0.0077 – 0.8784)***	**0.5476 (0.0999 – 0.995)***	0.1371 (-0.3372 – 0.6113)	0.3911 (-0.0824 – 0.8646)
**Pro-Inflammatory Cytokines**				
IFN-γ	-0.0038 (-0.1291 – 0.1215)	0.0615 (-0.818 – 0.2048)	-0.0565 (-0.1901 – 0.077)	0.0502 (-0.042 – 0.1424)
	0.0335 (-0.0901 – 0.157)	0.0552 (-0.0862 – 0.1967)	-0.0393 (-0.1736 – 0.095)	0.0412 (-0.0497 – 0.132)
TNF-α	1.001 (-0.8678 – 2.8698)	0.4221 (-1.3294 – 2.1735)	-0.0942 (-2.8349 – 2.6465)	0.3857 (-1.9783 – 2.7497)
	0.7949 (-1.0483 – 2.6381)	0.3924 (-1.3526 – 2.1373)	0.6591 (-2.0153 – 3.3336)	0.892 (-1.4126 – 3.1966)
IL-6	3.9587 (-0.6733 – 8.5908)	**4.9178 (0.1289 – 9.707)***	-0.0487 (-5.0883 – 4.9909)	1.3612 (-2.2633 – 4.9856)
	4.1484 (-0.4653 – 8.762)	4.9628 (-0.0001 – 9.9258)	1.6192 (-3.3397 – 6.5782)	1.7171 (-1.8928 – 5.327)
IL-18	-0.0021 (-0.0137 – 0.0096)	0.001 (-0.0092 – 0.011)	-0.0101 (-0.0232 – 0.0031)	-0.0062 (-0.0162 – 0.0037)
	-0.0017 (-0.0138 – 0.0104)	0.0019 (-0.0081 – 0.012)	-0.0105 (-0.023 – 0.002)	-0.0059 (-0.0156 – 0.0038)
**Pro-Inflammatory Chemokines**				
IL-8	0.6362 (-1.3244 – 2.5968)	-1.3959 (-3.9218 – 1.13)	1.2011 (-0.9631 – 3.3652)	0.751 (-1.1609 – 2.6628)
	0.3766 (-1.5617 – 2.3149)	-1.3615 (-3.8592 – 1.136)	1.609 (-0.4943 – 3.7126)	0.8559 (-1.8928 – 5.327)
IP-10	0.019 (-0.0099 – 0.048)	0.0248 (-0.0025 – 0.0521)	-0.0203 (-0.0537 – 0.013)	0.0123 (-0.0167 – 0.0412)
	0.0244 (-0.0044 – 0.0532)	**0.0275 (0.0001 – 0.055)***	-0.024 (-0.0572 – 0.0091)	0.0062 (-0.0222 – 0.0346)
MCP-1	0.0109 (-0.0443 – 0.0661)	-0.0052 (-0.067 – 0.0567)	-0.0103 (-0.0769 – 0.0563)	-0.0084 (-0.0836 – 0.0668)
	0.006 (-0.0483 – 0.0603)	-0.0135 (-0.076 – 0.049)	-0.0063 (-0.0714 – 0.0588)	-0.0025 (-0.0755 – 0.0705)
SDF-1 α	-0.0006 (-0.0085 – 0.0072)	-0.0015 (-0.009 – 0.006)	-0.007 (-0.0157 – 0.0016)	-0.0038 (-0.0126 – 0.005)
	-0.0006 (-0.0083 – 0.0072)	-0.0031 (-0.011 – 0.0045)	-0.0071 (-0.0155 – 0.0013)	-0.0024 (-0.0111 – 0.0062)
MIF	0.0435 (-0.0588 – 0.1457)	0.0765 (-0.0404 – 0.1933)	-0.0181 (-0.1365 – 0.1004)	-0.0138 (-0.1449 – 0.1173)
	0.0271 (-0.0731 – 0.1273)	0.0744 (-0.0405 – 0.1893)	-0.0221 (-0.1363 – 0.092)	-0.0205 (-0.1472 – 0.1062)
**Tryptophan Metabolism**				
Trp	-0.0005 (-0.0018 – 0.0007)	-0.0003 (-0.0015 – 0.001)	-0.0012 (-0.0026 – 0.0002)	-0.0007 (-0.0023 – 0.001)
	-0.0009 (-0.0021 – 0.0003)	-0.0008 (-0.002 – 0.0005)	-0.0005 (-0.002 – 0.0009)	-0.0006 (-0.0022 – 0.001)
Kyn	0.0082 (-0.035 – 0.0514)	-0.0002 (-0.0388 – 0.038)	-0.0302 (-0.0695 – 0.0091)	-0.0234 (-0.0718 – 0.025)
	0.0052 (-0.0382 – 0.0487)	-0.0018 (-0.041 – 0.0377)	-0.0099 (-0.0494 – 0.0296)	-0.0086 (-0.0561 – 0.0388)
Kyn/Trp	**456.6 (88.6 – 824.6)***	209.3 (-197.9 – 616.6)	-27.2 (-393.4 – 339.02)	-88.2 (-433.4 – 256.9)
	**583.6 (218.3 – 948.8)****	**481.5 (27.8 – 935.2)***	-50.6 (-419.5 – 318.3)	21.9 (-306.6 – 350.5)

Supplementary Table 5. Regression coefficients and confidence intervals are presented for linear models where STAI scores are regressed against biomarker levels both in isolation (rows with clear background) as well as adjusted for

age, BMI, socioeconomic index, smoking status and alcohol intake (rows with shaded background). Significant coefficients are highlighted in bold (* P<0.05; ** P<0.01; *** P<0.001).

	Healthy		IBS	
	**Week 15**	**Week 20**	**Week 15**	**Week 20**
	**β (95% CI)**	**β (95% CI)**	**β (95% CI)**	**β (95% CI)**
**Gut Permeability**				
IFABP	0.0002 (-0.0001 – 0.0004)	0.0003 (0 – 0.0006)	0 (-0.0003 – 0.0002)	0.0001 (-0.0002 – 0.0003)
	0.0002 (-0.0001 – 0.0004)	0.0003 (0 – 0.0006)	0.0001 (-0.0002 – 0.0003)	0 (-0.0002 – 0.0003)
LBP	0.1183 (-0.0128 – 0.2495)	**0.148 (0.0332 – 0.2632)***	-0.0019 (-0.1246 – 0.1209)	0.0167 (-0.0988 – 0.1321)
	0.1161 (-0.0136 – 0.2458)	**0.1355 (0.0217 – 0.249)***	0.0013 (-0.1199 – 0.1226)	0.0007 (-0.114 – 0.1153)
sCD14	0.0043 (-0.0003 – 0.008)	0.0039 (-0.0005 – 0.0083)	-0.0019 (-0.0058 – 0.002)	-0.0002 (-0.0045 – 0.0042)
	0.0032 (-0.0014 – 0.0078)	0.0026 (-0.0019 – 0.0072)	-0.0016 (-0.0053 – 0.0021)	-0.0002 (-0.0046 – 0.0041)
Anti-Endotoxin IgA	0.0004 (-0.0434 – 0.0441)	-0.0083 (-0.05 – 0.0335)	**0.0496 (0.006 – 0.0933)***	**0.055 (0.009 – 0.101)***
	-0.0048 (-0.0487 – 0.0392)	-0.0063 (-0.0483 – 0.036)	**0.0521 (0.008 – 0.0962)***	**0.0466 (0.0011 – 0.0921)***
Anti-Endotoxin IgG	0.0011 (-0.0183 – 0.0206)	-0.0055 (-0.025 – 0.0141)	0.0172 (-0.0007 – 0.0352)	0.0184 (-0.0023 – 0.0391)
	-0.0052 (-0.0252 – 0.0149)	-0.0055 (-0.025 – 0.014)	0.011 (-0.0066 – 0.0286)	0.0128 (-0.0079 – 0.0335)
Anti-Endotoxin IgM	0.0103 (-0.0213 – 0.0419)	0.0006 (-0.0334 – 0.0345)	0.0227 (-0.0054 – 0.0508)	0.0285 (-0.0021 – 0.0591)
	0.0065 (-0.026 – 0.0389)	-0.002 (-0.036 – 0.032)	0.0188 (-0.0083 – 0.0458)	0.0243 (-0.0054 – 0.054)
**Systemic Inflammation**				
CRP	0.1273 (-0.0679 – 0.3225)	0.1401 (-0.0566 – 0.3369)	-0.0102 (-0.1966 – 0.1763)	0.0491 (-0.1343 – 0.2325)
	0.1179 (-0.0767 – 0.3124)	0.1143 (-0.0856 – 0.3141)	0.0342 (-0.148 – 0.2163)	0.0925 (-0.09 – 0.2751)
**Pro-Inflammatory Cytokines**				
IFN-γ	-0.0223 (-0.0766 – 0.032)	-0.012 (-0.0744 – 0.0504)	-0.0335 (-0.0845 – 0.0175)	0.0154 (-0.02 – 0.0508)
	-0.0093 (-0.0638 – 0.0453)	-0.0125 (-0.074 – 0.0494)	-0.0264 (-0.0777 – 0.025)	0.0092 (-0.0256 – 0.0441)
TNF-α	0.35 (-0.4634 – 1.1634)	0.3978 (-0.3599 – 1.1555)	0.142 (-0.9091 – 1.193)	0.3703 (-0.5341 – 1.2748)
	0.2062 (-0.6087 – 1.0212)	0.314 (-0.4452 – 1.0732)	0.3116 (-0.7139 – 1.3371)	0.5047 (-0.3733 – 1.3827)
IL-6	0.038 (-2.0024 – 2.0785)	0.7396 (-1.3778 – 2.857)	0.866 (-1.0599 – 2.7919)	0.8236 (-0.5612 – 2.2085)
	-0.0574 (-2.1257 – 2.0109)	0.9197 (-1.281 – 3.1204)	1.2008 (-0.6906 – 3.0922)	0.7828 (-0.5948 – 2.1604)
IL-18	-0.0006 (-0.0056 – 0.0045)	0.0015 (-0.0029 – 0.0059)	-0.0022 (-0.0073 – 0.0028)	-0.0007 (-0.0045 – 0.0032)
	0.0002 (-0.0051 – 0.0056)	0.0021 (-0.0023 – 0.0065)	-0.0028 (-0.0076 – 0.0021)	-0.0008 (-0.0045 – 0.0029)
**Pro-Inflammatory Chemokines**				
IL-8	-0.1936 (-1.0462 – 0.659)	**-1.14 (-2.2208 – -0.0592)***	0.5813 (-0.246 – 1.4087)	0.4919 (-0.2374 – 1.2212)
	-0.2429 (-1.097 – 0.6112)	-0.992 (-2.0694 – 0.0854)	0.6275 (-0.1792 – 1.4342)	0.4493 (-0.2768 – 1.1754)
IP-10	0.0011 (-0.0116 – 0.0138)	0.0064 (-0.0056 – 0.0184)	-0.0116 (-0.0243 – 0.0011)	0.0057 (-0.0054 – 0.0168)
	0.0041 (-0.0088 – 0.0169)	0.0085 (-0.0036 – 0.0205)	-0.0117 (-0.0243 – 0.0009)	0.0044 (-0.0064 – 0.0152)
MCP-1	-0.0124 (-0.0363 – 0.0115)	-0.0155 (-0.042 – 0.0112)	0.0025 (-0.0231 – 0.028)	-0.0002 (-0.0291 – 0.0287)
	-0.0132 (-0.037 – 0.0106)	-0.0146 (-0.042 – 0.012)	0.003 (-0.022 – 0.028)	0.0019 (-0.026 – 0.0298)
SDF-1 α	-0.0023 (-0.0057 – 0.001)	-0.0014 (-0.005 – 0.002)	-0.0011 (-0.0044 – 0.0022)	-0.0008 (-0.0042 – 0.0026)
	-0.0021 (-0.0055 – 0.0013)	-0.0011 (-0.0044 – 0.002)	-0.0009 (-0.0042 – 0.0023)	0 (-0.0033 – 0.0033)
MIF	0.0325 (-0.0116 – 0.0766)	0.0147 (-0.0364 – 0.0648)	-0.0272 (-0.0724 – 0.0179)	-0.0152 (-0.0654 – 0.0351)
	0.0246 (-0.0193 – 0.0686)	0.0113 (-0.0392 – 0.0618)	-0.0254 (-0.0689 – 0.0182)	-0.0112 (-0.0596 – 0.0372)
**Tryptophan Metabolism**				
Trp	-0.0002 (-0.0007 – 0.0003)	0.0001 (-0.0004 – 0.0006)	-0.0004 (-0.001 – 0.0001)	-0.0004 (-0.0011 – 0.0002)
	-0.003 (-0.0008 – 0.0002)	0 (-0.0005 – 0.0006)	-0.0001 (-0.0007 – 0.0004)	-0.0003 (-0.0009 – 0.0003)
Kyn	-0.0041 (-0.0219 – 0.0137)	-0.0007 (-0.0166 – 0.015)	**-0.0176 (-0.032 – -0.003)***	-0.0165 (-0.0349 – 0.0019)
	-0.0045 (-0.0233 – 0.0143)	0.0001 (-0.0167 – 0.0169)	-0.0099 (-0.025 – 0.0052)	-0.0105 (-0.0291 – 0.0081)
Kyn/Trp	91.8 (-64.4 – 248.1)	-11.6 (-181.4 – 158.3)	-84.9 (-224.4 – 54.5)	-40 (-172.9 – 92.9)
	120 (-47.4 – 287.5)	58.3 (-140.5 – 257.1)	-106.6 (-246.9 – 33.8)	-4.8 (-134.5 – 125.7)

Supplementary Table 6. Regression coefficients and confidence intervals are presented for linear models where EPDS scores are regressed against biomarker levels both in isolation (rows with clear background) as well as adjusted for

age, BMI, socioeconomic index, smoking status and alcohol intake (rows with shaded background). Significant coefficients are highlighted in bold (* P<0.05; ** P<0.01; *** P<0.001).

Supplemental references

60. Grootjans J, et al. Non-invasive assessment of barrier integrity and function of the human gut. *World J Gastrointest Surg*. 2010;2(3):61–69.
61. Le Roy D, et al. Critical role of lipopolysaccharide-binding protein and CD14 in immune responses against gram-negative bacteria. *J Immunol*. 2001;167(5):2759–2765.
62. Maes M, et al. The effects of psychological stress on humans: increased production of pro-inflammatory cytokines and a Th1-like response in stress-induced anxiety. *Cytokine*. 1998;10(4):313–318.
63. Myint AM, et al. Tryptophan metabolism and immunogenetics in major depression: a role for interferon-γ gene. *Brain Behav Immun*. 2013;31:128–133.
64. Dunn AJ. Cytokine activation of the HPA axis. *Ann N Y Acad Sci*. 2000;917:608–617.
65. Carpentier PA, et al. Placental TNF-α signalling in illness-induced complications of pregnancy. *Am J Pathol*. 2011;178(6):2802–2810.
66. Rudolph MD, et al. Maternal IL-6 during pregnancy can be estimated from newborn brain connectivity and predicts future working memory in offspring. *Nat Neurosci*. 2018;21(5):765–772.
67. Maslanik T, et al. Commensal bacteria and MAMPs are necessary for stress-induced increases in IL-1β and IL-18 but not IL-6, IL-10 or MCP-1. *PLoS One*. 2012;7(12):e50636.
68. Liu YP, et al. Tumor necrosis factor-alpha and interleukin-18 modulate neuronal cell fate in embryonic neural progenitor culture. *Brain Res*. 2005;1054(2):152–158.
69. Fukuda H, et al. Stress assessment in acute care department nurses by measuring interleukin-8. *Int Nurs Rev*. 2008;55(4):407–411.
70. Shimoya K, et al. Interleukin-8 level in maternal serum as a marker for screening of histological chorioamnionitis at term. *Int J Gynaecol Obstet*. 1997;57(2):153–159.
71. Sun L, et al. Association between higher expression of interleukin-8 (IL-8) and haplotype −353A/−251A/+678T of IL-8 gene with preeclampsia. A case–control study. *Medicine (Baltimore)*. 2016;95(52):e5537.
72. Von Minckwitz G, et al. Predictive value of serum interleukin-6 and -8 levels in preterm labor or rupture of the membranes. *Acta Obstet Gynecol Scand*. 2000;79(8):667–672.
73. Gotsch F, et al. CXCL10/IP-10: a missing link between inflammation and anti-angiogenesis in preeclampsia? *J Matern Fetal Neonatal Med*. 2007;20(11):777–792.
74. Aminzadeh F, et al. Differential expression of CXC chemokines CXCL10 and CXCL12 in term and pre-term neonates and their mothers. *Am J Reprod Immunol*. 2012;68(4):338–344.
75. Asberg M, et al. Novel biochemical markers of psychosocial stress in women. *PLoS One*. 2009;4(1):e3590.
76 Oglodek EA, et al. The MCP-1, CCL-5 and SDF-1 chemokines as pro-inflammatory markers in generalized anxiety disorder and personality disorders. *Pharmacol Rep*. 2015;67(1):85–89.
77. Otsubo Y, et al. Association of cord blood chemokines and other biomarkers with neonatal complications following intrauterine inflammation. *PLoS One*. 2017;12(5):e0175082.
78. Guyan A. CXCL12 chemokine and its receptors as major players in the interactions between immune and nervous systems. *Front Cell Neurosci*. 2014;8:65.
79. Oglodek EA, et al. Comparison of chemokines (CCL-5 and SDF-1), chemokine receptors (CCR-5 and CXCR-4) and IL-6 levels in patients with different severities of depression. *Pharmacol Rep*. 2014;66(5):920–926.
80. Oglodek EA, et al. Serum concentrations of chemokines (CCL-5 and CXCL-12), chemokine receptors (CCR-5 and CXCR-4), and IL-6 in patients with posttraumatic stress disorder and avoidant personality disorder. *Pharmacol Rep*. 2015;67(6):1251–1258.
81. Petrovsky N, et al. Macrophage migration inhibitory factor exhibits a pronounced circadian rhythm relevant to its role as a glucocorticoid counter-regulator. *Immunol Cell Biol*. 2003;81(2):137–143.
82. Kim Y-K, Jeon SW. Neuroinflammation and the immune-kynurenine pathway in anxiety disorders. *Curr Neuropharmacol*. 2018;16(5):574–582.
83. Lanser L, et al. Inflammation-induced tryptophan breakdown is related with anemia, fatigue, and depression in cancer. *Front Immunol*. 2020;11:249.
84. Davis I, Liu A. What is the tryptophan kynurenine pathway and why is it important to neurotherapy? *Expert Rev Neurother*. 2015;15(7):719–721.
